# State-of-the-Art of Eggshell Waste in Materials Science: Recent Advances in Catalysis, Pharmaceutical Applications, and Mechanochemistry

**DOI:** 10.3389/fbioe.2020.612567

**Published:** 2021-01-27

**Authors:** Matej Baláž, Elena V. Boldyreva, Dmitry Rybin, Stefan Pavlović, Daily Rodríguez-Padrón, Tihana Mudrinić, Rafael Luque

**Affiliations:** ^1^Department of Mechanochemistry, Institute of Geotechnics, Slovak Academy of Sciences, Košice, Slovakia; ^2^Department of Solid State Chemistry, Novosibirsk State University, Novosibirsk, Russia; ^3^Boreskov Institute of Catalysis, the Siberian Branch of the Russian Academy of Sciences, Novosibirsk, Russia; ^4^Udmurt Federal Research Centre of the Ural Branch of the Russian Academy of Sciences, Izhevsk, Russia; ^5^Mezomax Inc., San Francisco, CA, United States; ^6^Department of Catalysis and Chemical Engineering, University of Belgrade – Institute of Chemistry, Technology and Metallurgy – National Institute of the Republic of Serbia, Belgrade, Serbia; ^7^Department of Organic Chemistry, University of Cordoba, Cordoba, Spain

**Keywords:** eggshell, eggshell membrane (ESM), mechanochemistry, catalysis, electrochemistry, biomedical applications, sustainable resources, waste treatment

## Abstract

Eggshell waste is among the most abundant waste materials coming from food processing technologies. Despite the unique properties that both its components (eggshell, ES, and eggshell membrane, ESM) possess, it is very often discarded without further use. This review article aims to summarize the recent reports utilizing eggshell waste for very diverse purposes, stressing the need to use a mechanochemical approach to broaden its applications. The most studied field with regards to the potential use of eggshell waste is catalysis. Upon proper treatment, it can be used for turning waste oils into biodiesel and moreover, the catalytic effect of eggshell-based material in organic synthesis is also very beneficial. In inorganic chemistry, the eggshell membrane is very often used as a templating agent for nanoparticles production. Such composites are suitable for application in photocatalysis. These bionanocomposites are also capable of heavy metal ions reduction and can be also used for the ozonation process. The eggshell and its membrane are applicable in electrochemistry as well. Due to the high protein content and the presence of functional groups on the surface, ESM can be easily converted to a high-performance electrode material. Finally, both ES and ESM are suitable for medical applications, as the former can be used as an inexpensive Ca^2+^ source for the development of medications, particles for drug delivery, organic matrix/mineral nanocomposites as potential tissue scaffolds, food supplements and the latter for the treatment of joint diseases, in reparative medicine and vascular graft producing. For the majority of the above-mentioned applications, the pretreatment of the eggshell waste is necessary. Among other options, the mechanochemical pretreatment has found an inevitable place. Since the publication of the last review paper devoted to the mechanochemical treatment of eggshell waste, a few new works have appeared, which are reviewed here to underline the sustainable character of the proposed methodology. The mechanochemical treatment of eggshell is capable of producing the nanoscale material which can be further used for bioceramics synthesis, dehalogenation processes, wastewater treatment, preparation of hydrophobic filters, lithium-ion batteries, dental materials, and in the building industry as cement.

## Introduction

Eggshell is one of the most common forms of food waste. Its production worldwide is 50,000 t per year (Palka, 2006). It basically consists of two parts: eggshell itself, which is mainly composed of calcium carbonate and eggshell membrane, which is a proteinous structure.

The chemical composition of eggshell has been reported many times (Nakano et al., 2003; Nys et al., 2004; Rose and Hincke, 2009). All reports agree that the main component is calcium carbonate in the form of calcite, its contribution is usually reported to be in the range of 94–97% (Burley and Vadehra, 1989; Stadelman, 2000; Hunton, 2005). The other constituents encompass Ca_3_(PO_4_)_2_ (1%), MgCO_3_ (1%), and organic material (4%) (Stadelman, 2000).

Apart from mainly inorganic ES, the eggshell membrane (ESM) is of purely organic character. It is composed of different proteins, the composition of which has been extensively discussed in the literature (Leach, 1982; Wong et al., 1984; Arias et al., 1991; Nakano et al., 2003; Zhao and Chi, 2009; Hincke et al., 2012; Kaweewong et al., 2013). The reported content of individual amino acids in eggshell membrane also varies in different literature sources. According to Nakano et al. (2003), the most abundant amino acids are proline, glutamic acid, and glycine.

The morphology of eggshell has been nicely described in Rodriguez-Navarro et al. (2007) and Zhou et al. (2010). It contains mainly calcite crystals, which are arranged into surface, palisade, and mammillary layers with different morphology and porosity. The eggshell membrane is a fibrous structure and the fibers are known to decrease in the diameter from outside of the egg to the inside (Zhou et al., 2010). They are also known to possess core-mantle structure, which differs in chemical composition (Li et al., 2011). The eggshell membrane can be subdivided into more structures- outer membrane, inner membrane and limiting membrane (Hincke et al., 2000). The sublayers of the ESM slightly differ in the individual amino acid content (Nakano et al., 2003). The largest difference has been observed in the case of leucine, which is more abundant in the outer ESM.

**Figure 1:**
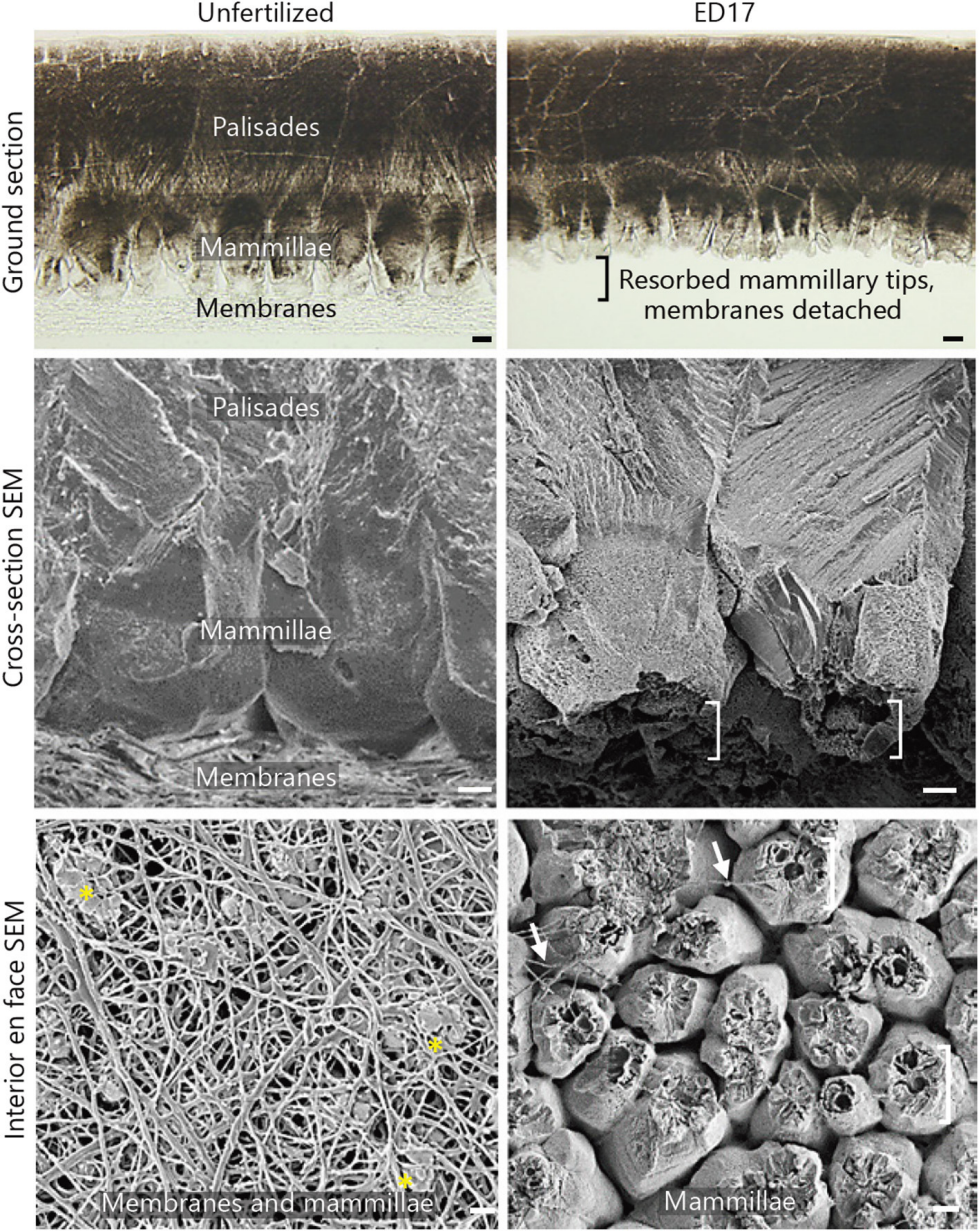
Ground and polished sections and scanning electron microscopy (SEM) images of the eggshell (ES) and its membranes in unfertilized (left) and fertilized egg (right), showing dissolution of the innermost portion of the ES and detachment of the membranes during incubation of the fertilized egg. Brackets- dissolved mammillary tips; asterisks- intact mammillary tips; arrows- residual membrane fibers. ED17 stands for day 17 of incubation. Scale bars, 10 μm (Hincke et al., 2019).

Although this paper is materials science-oriented, at least a short note about the function of eggshell and the membranes during the development of chicken embryo needs to be mentioned. Eggshell provides a barrier against pathogens and because of its porosity, it is permeable for gas exchanges. However, after fertilization, the eggshell starts to be degraded as the calcium is used for the proper development of the chick embryo. As a result of this, the bottom part of the mammillary layer is dissolved and eggshell membrane detaches (Hincke et al., 2019). This can be nicely seen from Figure 1, where the comparison of the morphology of ES/ESM of a fertilized and unfertilized egg is provided.

There are many review papers devoted to the eggshell waste showing their wide application potential in materials science (King'ori, 2011; Marwaha et al., 2018; da Silveira Pinto and de Souza, 2019; Konwar et al., 2019; Waheed et al., 2019, 2020; Girelli et al., 2020; Hamada et al., 2020; Hart, 2020). The use of eggshell waste as a food supplement (Waheed et al., 2019), for organic synthesis (da Silveira Pinto and de Souza, 2019), catalysis (Tan et al., 2015a; Laca et al., 2017), and adsorption (Carvalho et al., 2011; Guru and Dash, 2014; He et al., 2016) has been already reviewed in the past. Some technologies applying eggshell are patented (Balassa, 1971; Dawson, 2003; Cordeiro and Hincke, 2011; Schmidt et al., 2017; Kenny et al., 2018; Blaine and Thang, 2019; Huang et al., 2020). The proteinous eggshell membrane also has a great potential to be applied in materials science (Baláž, 2014), and its soluble form is applicable for tissue engineering (Sah and Rath, 2016). This contribution aims to shed a light on recent achievements in using eggshell waste in the field of materials science, namely targeting the fields of catalysis, electrochemistry, and medical applications. In the last part, an update on the recent publications utilizing mechanochemical approach for the treatment of eggshell waste is provided.

## Eggshell in Catalysis

Due to the remarkable importance of bioactive compounds for the chemical, pharmaceutical, cosmetic, and food industries, finding environmentally friendly synthetic strategies involving catalytic steps represents a key task for a sustainable development. In this regard, several factors need to be considered, including not only the green character of the catalytic materials but also the environmental benefits and the cost and energy efficiencies of the synthetic methodology for catalysts preparation. Biomass transformation toward advanced nanocatalytic systems has been demonstrated to be a very valuable option, which still requires further efforts in order to achieve optimized protocols and highly versatile and appealing materials. The suitable chemical composition, structural, morphological, and textural properties of eggshell are important to promote different catalytic reactions. Depending on the desired applications field, eggshell could be treated using different methods to obtain a catalyst with well-defined properties. The eggshell treatment methods may be divided into simpler ones (preceded by washing, cleaning, and drying), which include only thermal treatment (calcination in a muffle furnace in the oxygen or inert atmosphere in the temperature range 600–1,000°C) or more complex methods, which include chemical reaction, hydration, dehydration, precipitation, co-precipitation, deposition, sol-gel, and interaction between two components (one of which is mainly catalytic support). In order to avoid some toxic salt solutions and save energy, there are some waste-free and low-energy demanding techniques for catalyst synthesis, such as mechanochemical one. It will be shown below that the attention of many researchers is focused on the utilization of eggshell in different environmentally beneficial processes.

### Synthesis of Bioactive Compounds and the Eggshell-Based Catalysis in Organic Synthesis

Eggshell-derived nanocatalysts have been reported for the synthesis of various bioactive compounds, such as chromenones, pyran derivatives, polyhydroquinolines, aromatic aldehydes, benzothiazoles, and carbohydrates (Laca et al., 2017).

*N*-heterocycles, such as nitrogen-containing phthalazine compounds, could have potential applications as antimicrobial, antioxidant, and anti-convulsant agents. The synthesis of this family of compounds is achieved through a multicomponent reaction employing heterogeneous catalytic systems. For instance, the use of eggshell powder as a catalytic material for the synthesis of pyrazolo-phthalazine derivatives via four-component condensation through a Knoevenagel–Michael reaction has been reported. Remarkably, the aforementioned reaction was performed in water, as a green solvent, and under moderate conditions, in terms of temperature and reaction time. Under the aforementioned parameters, eggshell powder exhibited good catalytic performance, achieving 93–98% yield of the desired products. More importantly, as one of the most valuable possibilities that heterogeneous catalysis offers, recyclability of the material was carried out for four cycles without a considerable loss of activity (Kerru et al., 2020). Moreover, spiro-heterocycles, due to their biological activity, could also be employed for a wide range of applications as analgetic, fungicidal, and antibacterial agents. Such kind of heterocycles have been prepared by a three-component reaction, passing as well through a Knoevenagel condensation and a Michael addition, which require a catalytic system. In this sense, milled eggshell has served as an active catalyst, displaying both good catalytic activity and stability over five reaction cycles (Youseftabar-Miri, 2019). Milled and calcined eggshell residues have been also used for Knoevenagel condensation of aromatic aldehydes, which could give rise to a myriad of organic compounds with pharmacological features, such as benzylidenepropanedinitrile (Patil et al., 2013). Similar eggshell-based materials have been demonstrated to be potential candidates as catalytic materials for benzothiazoles preparation. These molecules are important bioactive scaffolds with antitumor, antiallergic, antidiabetic, and antimicrobial agents (Borhade et al., 2016).

In the aforementioned cases, the catalysts were prepared by simply milling, usually using a mortar and pestle, which certainly are one of the least expensive tools in mechanochemistry. However, the use of such instruments possesses several disadvantages, since it is not possible to precisely control milling conditions and therefore material reproducibility is compromised. Looking forward to overcoming these issues, several reports in the literature have considered the use of ball milling for the preparation of similar materials. In this sense, the ball milling assisted synthesis of nano-CaO derived from eggshell residues for the solventless preparation of pyrano[4,3-b]pyran derivatives has been reported. Such a catalyst-design strategy has a strong sustainable character, since it combines the use of a readily available waste, as CaO precursor, with the employment of a solvent-free preparation procedure. This study also considered the effect of the mechanochemical approach in the CaO particle size and consequently in the catalytic performance, revealing that longer milling times resulted in smaller catalyst particle size and higher product yield (Mosaddegh and Hassankhani, 2014). Mechanochemical methods have also been employed for the preparation of nano-bio calcite (CaCO_3_), employing eggshell as a green source. Such material has demonstrated to be an efficient catalytic system, in terms of activity and reusability, for the solvent-free synthesis of pyrano[4,3-*b*]pyrans at 120°C. Outstandingly, the eggshell derived material displayed higher surface area and improved catalytic results, in comparison with the commercially available CaCO_3_, validating the catalyst design and the efficient use of eggshell as CaCO_3_ precursor for organic synthesis applications (Mosaddegh et al., 2013).

Besides mechanochemistry, also other methodologies have been employed for the synthesis of eggshell derived materials, including ultrasound treatment and impregnation approaches. For instance, nano-eggshell powder has been prepared through an ultrasound-assisted procedure in a CH_2_Cl_2_ solution. The catalytic behavior of the obtained sample was evaluated in the thermal-assisted solventless condensation reaction of α- or β-phathol, malononitrile, and aromatic aldehydes, to obtain 2-aminochromenes, showing high product yields in short reaction times. 2-aminochromenes possess a wide range of applications due to their antioxidant, antiviral, anti-tubulin, antidepressant, and antihypertensive activities, among others. The sonochemically prepared nano-eggshell powder exhibited enhanced catalytic performance in comparison with the ultrasound-treated CaCO_3_ (Mosaddegh, 2013).

Eggshell as a catalyst for base-catalyzed reactions can be also applied for the production of important industrial chemicals, such as dimethyl-carbonate, oximes, and glycerol oligomers. In order to avoid some toxic chemicals, such as dimethylsulfate and methylhalides in methylation reactions and phosgene in polycarbonate and isocyanate synthesis, dimethylcarbonate presents a suitable chemical that meets many aspects of sustainable and green chemistry (Sankar et al., 2010). In Gao and Xu's (2012) study, the successful dimethylcarbonate synthesis was performed by the reaction between propylenecarbonate and methanol in the presence of calcined eggshell as a catalyst. In this reaction, the catalyst exhibited high activity (achieved yield was 75%) and suitable stability (five reaction cycles). Despite the presence of impurities (Mg and P) in the final catalyst, the catalytic activity remained unchanged. Another group of compounds important for various organic syntheses (preparation of nitriles, amines, nitro compounds, paracetamol, oxime ethers, azatricyclic core of (±)-halichlorine, and amides), which may be obtained in the process catalyzed with eggshell, are oximes. Using eggshell-based catalyst prepared by alkali and alkali-thermal treatment, oximes yield increased from 17 to 20 times (depending on the conditions of the treatment procedure), in comparison with the non-catalyzed process, respectively (Taleb et al., 2017). The suitable catalytic activity with prepared catalysts was achieved even after seven cycles, whereas the activity drop was slightly larger for alkali-thermal treated eggshell than for only alkali-treated one. The eggshell catalyst was also helpful in the reaction of glycerol-oligomers production, as its presence led to the 3,5-fold increase of reaction yield for glycerol conversion and 1.5-fold increase for oligomers yield in comparison with the non-catalyzed process (Barros et al., 2017). However, due to high calcium leaching in the reaction mixture at reaction conditions used (catalyst loading of 2 wt% and temperature of 220°C), the catalyst exhibits low stability.

### Wastewater Treatment: Opportunities in Photocatalysis

**Figure 2:**
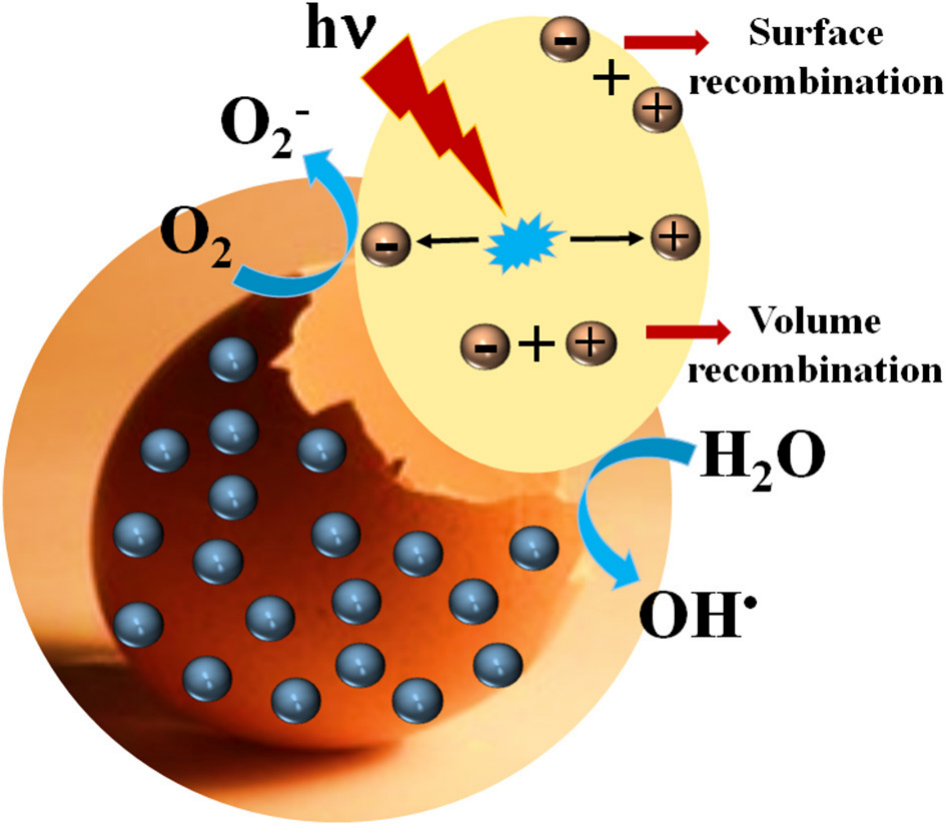
Schematic illustration of photocatalytic processes on the eggshell-modified surface (blue spheres correspond to photocatalytically active semiconductor nanomaterials).

Eggshell-supported semiconductor materials have also been employed for photocatalysis, which is an area of catalysis with an intrinsic environmentally friendly nature, where the rate of the chemical reaction is driven by the absorption of light irradiation by a semiconducting material (photocatalyst) (Rodriguez-Padron et al., 2020). Eggshell residues have been employed either as support of semiconductor materials, or as a biotemplate for the synthesis of semiconductor samples. As shown in Figure 2, the absorption of ultraviolet, visible or infrared radiation gives rise to the generation of holes and electrons, which could initiate oxidative and reductive reaction pathways, respectively (Rodriguez-Padron et al., 2019). However, surface or volume recombination phenomenon could occur, diminishing the photocatalytic activity of the samples. The efficiency of the photocatalytic process will depend on the semiconductor material. It should be also highlighted that when looking for more sustainable protocols, semiconductors able to absorb visible light are highly desirable (Munoz-Batista et al., 2018). Wastewater treatment is one of the main applications of photocatalytic processes, which could not only result in the degradation of pollutants but also in their transformation into valuable chemicals. Nowadays, the presence of dyes and organic compounds in wastewater from the pharmaceutical and textile industries constitute a major concern and an ecological challenge. For instance, the presence of dyes in wastewater could negatively affect the photosynthetic function of plants, and aquatic life by decreasing light penetration and oxygen consumption (Holkar et al., 2016).

**Figure 3:**
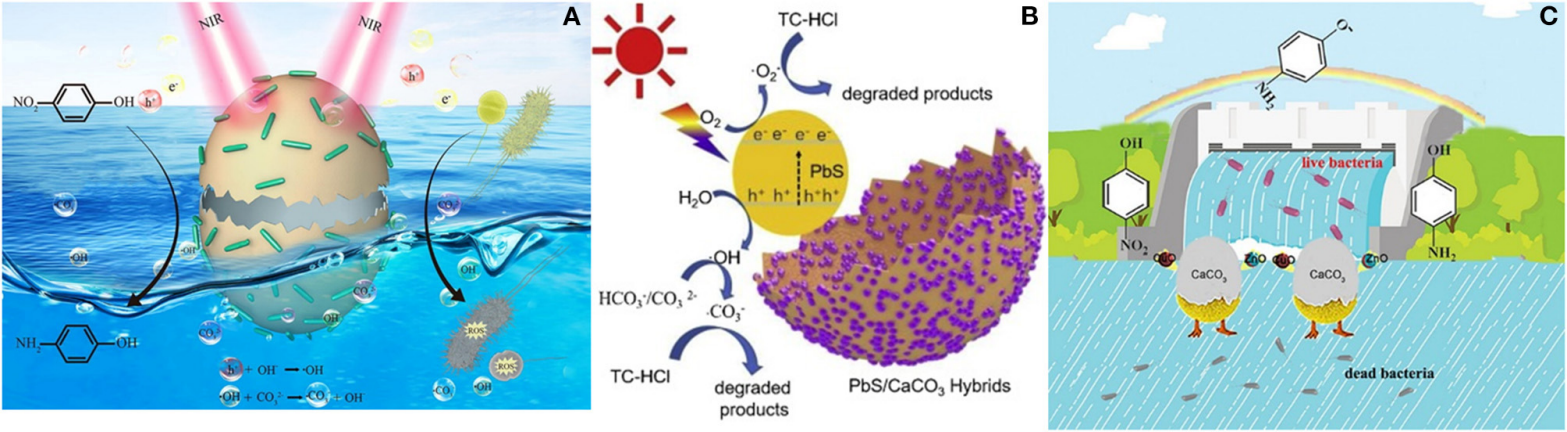
**(A)** Schematic representation of 4-NP degradation using CaCO_3_/CuS. Reprinted from Zhang et al. (2020b), Copyright (2020) American Chemical Society; **(B)** Illustrative degradation of TC-HCl over PbS/CaCO_3_ catalyst. Reprinted from Zhang et al. (2020a), Copyright (2020), with permission from Elsevier; **(C)** Schematic representation of 4-NP degradation using CuO/ZnO/eggshell. Reprinted by permission from Springer Nature Customer Service Centre GmbH: Springer Nature (Zhang et al., 2019a), Copyright (2019).

Among the possible semiconductors that could be employed in photocatalytic applications, sulfides have risen as suitable materials for several reactions. For instance, various transition-metal sulfides, including ZnS, Cu_2_S, CdS, In_2_S_3_, WS_2_, and MoS_2_, have been reported as effective photocatalysts (Rodriguez-Padron et al., 2020). In this sense, Luque et al. have developed an efficient photocatalytic system based on copper sulfide and calcium carbonate, employing eggshell waste as a well-known source of CaCO_3_ (Zhang et al., 2020b). In this work, eggshell is employed as a sustainable support, allowing the proper deposition of CuS nanoparticles, avoiding possible sintering effects, as indicated in the SEM-mapping analysis and facilitating the recovery and reuse of the sample. In addition, the presence of CaCO_3_ also resulted in the formation of reactive ^•^CO_3_^−^ species, which participate in the catalytic processes. CaCO_3_/CuS nanocomposites were prepared by an impregnation method, after eggshell residues, used as a template, were powdered and homogenized. The obtained samples were tested in the Near-infrared (NIR) light (according to their absorption in the NIR and visible regions) induced photocatalytic degradation of 4-nitrophenol (4-NP), which is a typical water pollutant and could cause some alterations in human endocrine systems (Zhang et al., 2020b) (Figure 3A). The reduction of 4-NP gives rise to the formation of 4-aminophenol (4-AP), which in turn is a valuable intermediate in the chemical industry, with applications in the production of valuable materials, such as analgesic and antipyretic drugs and anti-corrosion substances.

Lead sulfide is another semiconductor material, which has also been used, in combination with calcium carbonate from eggshell wastes, for solar light assisted photo-degradation of tetracycline hydrochloride (Zhang et al., 2020a). Interestingly, this work provides insights into the photocatalytic reaction mechanism of carbonate-based composite materials, where besides ^•^O_2_^−^, ^•^CO_3_^−^ also acts as an active species. These results could be understood considering that PbS excitation gives rise to electron-hole pairs, and particularly the generated holes participate in the oxidation of H_2_O into ^•^OH. Such radicals react with carbonate and bicarbonate ions to form ^•^CO_3_^−^(Zhang et al., 2020a) (Figure 3B).

Eggshell residues modified with polyethyleneimine (PEI), have also been used as support for the deposition of titania (TiO_2_) nanoparticles (Li et al., 2017). The modification with PEI provided amine groups on the surface of the eggshell membrane, which further favors the interactions with TiO_2_. The obtained material displayed good photocatalytic behavior for the degradation of pollutants, in particular Rhodamine B, which is a common dye in wastewater (Li et al., 2017). In addition, ZnO-based materials have been broadly studied for photocatalytic applications, as an alternative to titania. The similar band gap energy, together with the higher absorption efficiency under sunlight irradiation of ZnO (in comparison to TiO_2_) open up new possibilities for its application for visible light photocatalysis. Eggshell residues have been also used as support for ZnO semiconductor material. For instance, Danish et al. have reported the preparation of ZnO-CuO-supported on eggshell for photocatalytic degradation of dyes and organic compounds (Khairol et al., 2019a). The designed material displayed good results for the degradation of methylene blue, congo red, and phenol, among others, suggesting that it could be effectively used for wastewater treatment (Khairol et al., 2019a,b).

Nanocomposite materials based on ZnO and CuO employing eggshell residues as biotemplate have also been reported and tested as potential photocatalytic systems for the reduction of 4-nitrophenol. Remarkably, the aforementioned pollutant could be completely degraded by using CuO/ZnO/eggshell sample, within 8 min under light irradiation, most likely due to the efficient charge separation and the good absorbance features. Moreover, the catalyst exhibited a good stability over 5 reaction cycles, demonstrating to be a sustainable and effective candidate for photocatalytic degradation of pollutants (Zhang et al., 2019a) (Figure 3C). Besides their application for 4-NP reduction, similar materials have been also used for the photocatalytic degradation of methyl orange, achieving excellent results (He et al., 2019).

Furthermore, Yassein et al. have investigated the reduction of 4-NP toward 4-AP using CuO-eggshell nanocomposites (Sajadi et al., 2018). In this case, the effect of NaBH_4_ in the reaction media was also studied, indicating that the reaction proceeds with the absorption of 4-NP and hydrogen over the metal surfaces, electron transfer from ${\text{BH}}_{4}^{-}$ to 4-NP through CuO and final desorption of 4-AP. More importantly, CuO-eggshell nanocomposites displayed better catalytic performance in comparison with pure copper oxide, also indicating a synergistic effect between CuO nanoparticles and eggshell component (Sajadi et al., 2018).

Moreover, calcium oxide nanoparticles derived from eggshell residues have also been employed as a catalytic material for the degradation of dyes, including methylene blue and toluidine blue. In this regard, Rajasekaran et al. performed a parametric analysis, considering several factors affecting the degradation rate such as catalysts loading, dye concentration and pH (Vanthana Sree et al., 2020). The kinetics of dye degradation over eggshell derived CaO material was also investigated in this work, revealing that the reaction followed a pseudo-first order. In addition, the scavenging analysis was performed for methylene blue degradation, indicating that ^•^${\text{O}}_{2}^{-}$ are the primary active species in the aforementioned photocatalytic process. The eggshell-derived material exhibited outstanding stability within seven reaction cycles, demonstrating to be an excellent candidate for wastewater treatment and dye degradation (Vanthana Sree et al., 2020). Similarly, CaO nanocrystals derived from eggshell residues by thermal annealing have been used for the sunlight-assisted photocatalytic degradation of indigo carmine. In this work, the authors further modified the calcium oxide material with silver nanoparticles. However, no considerable differences were observed for both materials (CaO nanocrystals and Ag@CaO composite) in the photocatalytic dye degradation. In any case, unmodified calcium oxide nanocrystals exhibited a noticeable photocatalytic activity, with percentages of dye degradation around 99% (Alsohaimi et al., 2020).

### Other Environmentally Beneficial Applications in Catalysis

**Figure 4:**
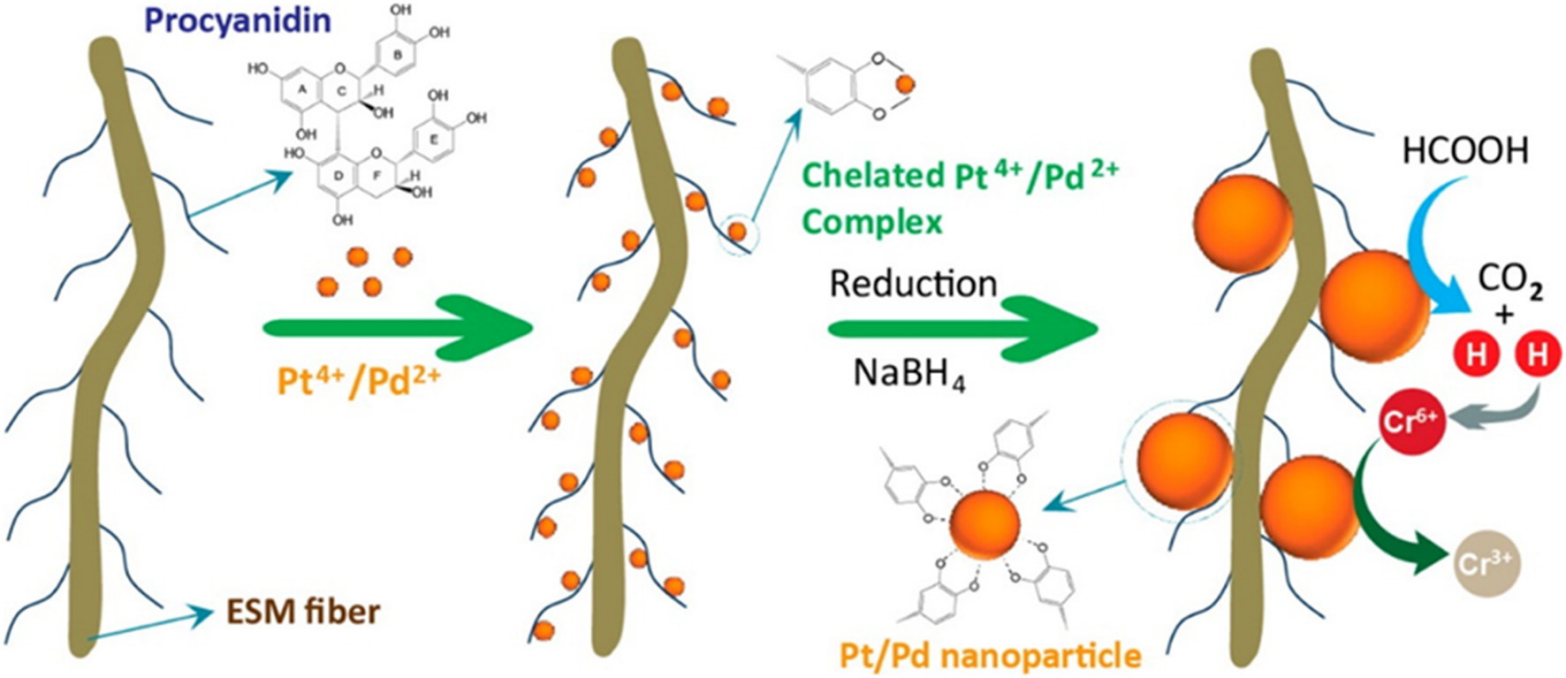
Synthetic strategy for bionanocomposites based on eggshell and noble metal (Pt, Pd) nanoparticles and their application for Cr (IV) reduction. Reprinted from Liang et al. (2014), Copyright (2014) American Chemical Society.

Besides all the possibilities of the use in catalysis mentioned so far, eggshell-derived materials can be also applied in a conventional heterogeneous catalysis. For instance, bionanocomposites based on eggshell as a bioscaffold and noble metal (Pt, Pd) nanoparticles have been synthesized and used for catalytic reduction of Cr(VI) to Cr(III) in aqueous solution, employing formic acid as a reducing agent (Figure 4). Importantly, metal nanoparticles were found to be well-dispersed and maintained good stability on the eggshell based support. It is well-known that Cr(VI) species are serious environmental pollutants, which are present in wastewater from several industries. Indeed, hexavalent chromium could have mutagenic and carcinogenic effects on human health. In turn, chromium (III) species are much less toxic and are used to form insoluble hydroxides. Therefore, the reduction of Cr(VI) into Cr(III) is a promising strategy for water remediation and this could be achieved using Pt/Pd nanoparticles embedded on the eggshell membrane (Liang et al., 2014).

Eggshell-derived materials functionalized with copper and iron oxide at different concentrations have been also designed for the application in the catalytic wet oxidation of humic acid (a refractory compound present in industrial wastewaters) (Oulego et al., 2020). In this work, it was found that copper-modified eggshell showed enhanced catalytic activity in comparison with unmodified eggshell, most likely due to the better metal distribution on the eggshell support. Interestingly, eggshell by itself could also be employed to degrade humic acid and, even if less effectively, this option should be considered for future works due to the cost-efficiency of the catalyst design (Oulego et al., 2020). In another example, Asgari and coworkers have reported the preparation of carbon-doped magnesium oxide on eggshell powder as an efficient catalyst for the ozonation process to treat real wastewater from the textile industry, displaying improved results in comparison with commercially available activated carbon (Asgari et al., 2019).

In summary, eggshell-derived samples have a broad range of opportunities in catalytic and photocatalytic processes for wastewater treatments. Such materials include calcium oxide, calcium carbonate or even eggshell without exhaustive treatments. In particular, for photocatalytic reactions employing calcium carbonate, it has been demonstrated that ^•^CO_3_^−^ species also participate in the reaction mechanism. The eggshell-based catalysts could act either as efficient support or active species. Eggshell residues certainly represent an outstanding alternative with unlimited opportunities to materials traditionally applied in catalysis.

### Eggshell as a Biodiesel Synthesis Catalyst

#### The Importance of Pretreatment

It is evident that calcium oxide represents a promising catalyst for biodiesel production due to many advantages (high catalytic activity, low solubility in methanol, non-toxicity, low price, availability in nature, and being a waste material) (Marinkovic et al., 2016; Marwaha et al., 2018). In the case of eggshell utilization to obtain active catalysts, the starting material was mainly thermally treated. Namely, in the synthesis process, the pre-treatment stage is very important due to the fact that eggshell waste contains different impurities, such as the eggshell membrane, which must be removed before the calcination process. The eggshell membrane can be partially carbonized during the calcination process, whereby the obtained carbon can strongly influence the development of the porous network system inappropriate for large organic molecules, such as triacylglycerols. The calcination process is carried out mainly in the muffle furnace in the temperature range between 850 and 950°C in different atmospheres (N_2_ and O_2_ both static and flow) (Salaudeen et al., 2018; Lima and Perez-Lopez, 2020), where a complete conversion of carbonate into oxide form can be achieved. Calcination conditions affect the textural properties, especially the specific surface area. In (Lima and Perez-Lopez, 2020), it was shown that the low specific surface area of the raw eggshell (1.3 m^2^ g^−1^) was increased almost 10 times by calcination in the flow of nitrogen. The specific surface area increase (albeit about five times smaller) was also achieved using calcination in the flow of oxygen. On the other hand, calcination of eggshell in the static oxygen atmosphere did not affect the specific surface area at all. Additionally, the study (Salaudeen et al., 2018) showed that calcination in the flow of carbon-dioxide is very slow and leads to incomplete conversion (max. 21 wt%) of calcium carbonate into calcium oxide due to the increase of CO_2_ partial pressure. Many studies showed that eggshell-derived calcium oxide exhibits high catalytic activity. However, low specific surface area, a non-uniform basic active sites distribution, non-defined porous network of pure and only partly calcined calcium oxide lead to low catalytic activity. Also, only partly calcined eggshell exhibits a higher tendency to leaching into reaction mixture, whereby catalyst becomes non-stable and unusable in the next reaction cycles. In order to avoid such undesirable behavior, more complex synthesis procedure should be used. Better textural, morphological, and basic properties of calcined eggshell can be obtained by hydration-dehydration (Yoosuk et al., 2010) or impregnation of calcium oxide on a suitable support, whereby it is important that suitable interaction between active species and support is achieved.

#### Biodiesel Production Results

The study (Pavlovic et al., 2020) showed that pure calcined eggshell exhibits great catalytic potential in biodiesel production. The catalytic activity and stability increased especially if the calcium oxide is dispersed over catalytic support, whereby the leaching of the active species is significantly reduced. In the same study, it was shown that pure calcined eggshell exhibited almost four times higher leaching of calcium into the crude biodiesel than coal fly ash-supported chicken eggshell calcium oxide. The catalyst was stable even after five reaction cycles with a negligible drop in catalytic activity. In the case when feedstock for biodiesel production contains some waste oils with high free fatty acid content, calcium oxide-supported catalysts represent satisfactory solution due to a suitable interaction between calcium oxide, as active component and the support (Stanković et al., 2020). Particularly, the role of support becomes more dominant when it exhibits an acidic character due to the presence of acid sites. Such catalysts exhibit bifunctional behavior by simultaneously carrying out the reactions of esterification and transesterification.

The studies by Gupta and Rathod (2018) and Gollakota et al. (2019) investigated the behavior of calcium oxide-supported catalysts in biodiesel synthesis from waste cooking oil. High conversion (>93.1%) and biodiesel yield (>96.3%) were obtained at relatively mild reaction conditions without the need of feedstock pretreatment using two eggshell-supported catalysts (calcium diglyceroxide and pyrolysis residue). In the case of pure calcined eggshell under the same operating conditions conversion (82.1%) and biodiesel yield (72.3%) were lower. A significant catalytic activity of eggshell in the production of biodiesel from waste cooking oil under solar irradiation was evidenced in the research of Bharti et al. (2020). It should be noted that despite lower reaction temperature (40–46°C), which is below the usual (≈60°C), and waste feedstock used (waste cooking oil), eggshell-based catalyst exhibited relatively high conversion (≈90%) in the short reaction time (2 h). In addition, the catalyst was stable and was successfully re-used in three reaction cycles. Calcium oxide from eggshell exhibits high catalytic potential for the biodiesel synthesis from the highly acidic feedstock, like chicken fat. However, such feedstock needs to be first esterified to adjust free fatty acid content to be lower than 4%. Kirubakaran and Selvan (2018) have shown that eggshell calcined at 900°C was still active after five reaction cycles with the highest biodiesel yield of 85% (Kirubakaran and Selvan, 2018).

### Gasification Processes

The simpler preparation procedure was used for utilization of eggshell in biomass or coal thermal conversion, which leads to the formation of various gases (depending on biomass and coal type). It can be seen that previously mentioned pre-treatment methods and calcination are sufficient to obtain a final active catalytic form. In these reactions, selectivity is particularly important. The study by Raheem et al. (2019) investigated eggshell-catalyzed gasification of algal biomass. During the catalyst-free process, the undesirable reactions, such as cracking and combustion occurred, and the composition of generated gas was shifted toward CO and CO_2_ with a small quantity of C_2_H_2_ and CH_4_. On the other hand, the presence of calcined eggshell led to the production of hydrogen, the amount of which increased with an increase in catalyst loading. This can be explained by adequate CO_2_ sorption properties of calcium oxide. The gasification process in the presence of calcium oxide takes place in two stages (rapid chemical reaction and diffusion controlled regime) and the carbonation at the first stage is inhibited by the growth of the calcium carbonate layer (Salaudeen et al., 2018). The low particle size of calcium oxide enhances the carbonation process, due to the more accessible surface. The main by-products, which can deactivate the catalyst are tar and char. Calcium oxide's ability to absorb CO_2_, the benefits of which are reflected in gasification reaction and lead to a selective gas formation, was used in the steam gasification process of low-rank coal, such as the sub-bituminous one used in Fan et al. (2017). This study shows the success of using the eggshell as a catalyst, as it improves carbon dioxide conversion and yields of hydrogen and carbon monoxide in the syngas. Favorable calcium behavior in the gasification reaction is explained by Ohtsuka and Asami (1997), who stated that calcium ions first undergo the ion-exchange process with carbonyl group present in coal due to their high basicity. The main role of dispersed calcium is reflected in its strong interaction with carbon, which leads to the dissociation of oxygen-containing gas and oxygen spillover with the formation of oxygen complexes as active catalytic sites responsible for successful performance the coal gasification reaction.

### Catalytic Oxidation

In these processes, pure eggshell does not exhibit activity at oxidation temperature characteristic for processes with conventional catalysts. However, eggshell exhibits better catalytic support in comparison with commercial calcium carbonate and allows better interaction with active species. The recent investigations show that eggshell-based oxidation catalysts exhibit acceptable activity with a special emphasis on activity, stability, and selectivity. In the study by Li et al. (2020), the eggshell-supported Co_3_O_4_ catalyst for benzene oxidation has been prepared. The catalyst was synthesized by the impregnation method using Co acetate solution, and the obtained precursor was calcined at different temperatures (300–500°C). This process resulted in the uniform Co_3_O_4_ nanoparticles distribution on the eggshell support. Comparing with benzene oxidation with pure eggshell, where oxidation was carried out at 380°C the oxidation with eggshell-supported Co_3_O_4_ catalyst could be performed at a lower temperature (256°C), yielding the same result. Also, the concentration of Co_3_O_4_ above 16.7% did not contribute to further activity increase. By *in situ* FTIR analysis, it is determined that active oxygen species in the catalyst directly participate in the reaction. The prepared catalyst was stable with constant benzene conversion of 95% during 50 h. Similar research was conducted by Guo et al. (2019) with eggshell-supported Ag catalyst. It was determined that optimal Ag concentration for this process was 19.9%, whereas the necessary oxidation temperature was lower (225°C) than in the case of Co_3_O_4_/eggshell-catalyzed process. It is important to note that this catalyst was stable even after 200 h with achieved benzene conversion of 95%. In the preparation methods of oxidation catalysts, it can be seen that the eggshell membrane is not removed, which is contrary to the preparation methods of other catalysts. It has been confirmed that eggshell membrane is very important in regulating the particle size and metal distribution due to strong metal-protein bonding interactions. Unlike the benzene oxidation process, pure eggshell catalyst exhibits suitable activity in oxidative coupling of methane to light olefins (Lima and Perez-Lopez, 2020). In the case of catalyst, the main role is played by specific surface area, which was controlled using different calcination conditions [e.g., the changing the calcination atmosphere (N_2_ or O_2_) and its state (static or flow)]. Catalysts obtained in the flow N_2_ and O_2_ atmosphere have shown similar methane conversion (30% at 800°C), whereas the catalyst calcined in static air exhibited the lowest activity due to the low specific surface area. On the other hand, using catalysts calcined in the flow N_2_ or O_2_, equal amounts of ethane and ethylene fractions were generated, which is not case for the catalyst obtained by calcination in static air (more selective toward ethane than ethylene). At a high reaction temperature (800°C), the eggshell catalysts calcined in flow of N_2_/O_2_ (methane conversion of 25%) and those calcined in static air atmospheres (methane conversion of 17%) were stable for 5 h.

These studies have demonstrated that eggshell-based catalysts outperformed other types of support materials (i.e., commercial CaCO_3_; Guo et al., 2019; Li et al., 2020 and oyster shell; Li et al., 2020) as well as pure NPs (Guo et al., 2019; Li et al., 2020). Moreover, Guo et al. showed that Pt NPs/eggshell exhibited a more preferable catalytic activity compared to that of commercial 5Pt/C catalyst (Guo et al., 2020). Authors working in this field claim that the enhanced catalytic activity of eggshell-based catalysts is driven by the comprehensive effect of chemical composition and morphology of the eggshell. More specifically, functional groups present on the surface of the eggshell provide a high dispersion of NPs on eggshell supports through the strong metal-functional groups bounding interaction and consequently improved catalytic performances. The strong interaction between NPs and eggshell causes the decomposition of the eggshell (i.e., CaCO_3_) to take place at a lower temperature compared to that of the pure eggshell, which has a positive impact on catalytic performance. The hierarchical porous structure of eggshell also significantly contributes to the enhancement of overall catalytic performance as it increases the contact area between VOCs and NPs and enhances mass and energy transfer. Finally, it is worth mentioning that the fine-tuning of catalytic performance [i.e., turnover frequencies and the temperature for achieving 90% benzene conversion (T_90%_)] of eggshell-based catalysts can be easily accomplished by the appropriate choice of parameters such as type of metal, metal loading, method of synthesis, calcination atmosphere, and temperature (Guo et al., 2019, 2020; Li et al., 2020).

### Limitations

Despite many advantages of the eggshell (availability, non-toxicity, and low price), there are certain limitations in terms of textural properties that complicate the process of eggshell application and manipulation. These limitations are reflected in an insufficiently developed system of pores and channels, characteristic for non-porous materials, materials with macropores or open voids, that are directly related to the active specific surface (Tan et al., 2015b). Moreover, the specific surface area is in this case correlated with the distribution of active species available for the chemical reaction, which has a direct impact on catalytic activity and hence the higher surface area catalyst is expected to have higher catalytic activity (Kumar and Ali, 2012). However, during catalyst preparation, it is important to conduct a controlled synthesis in order to obtain an optimal specific surface area with a well-developed pores in the mesoporous region. Special attention should be drawn to the organic part of the eggshell, which can be transformed into unburned carbon, which leads to a formation of material with high specific surface area and pores in the micro-region (Manique et al., 2017). The biodiesel production is strongly influenced by intra-catalyst mass transport limitations, which are due to the slow diffusion of high molecular weight triacylglycerol molecules inside the catalyst process (Pavlovic et al., 2020). This is precisely the reason why it is important to make eggshell modification in terms of adjusting the specific surface area, and obtaining a system of available pores and channels with the adequate positioning of the active catalytic species.

## Application of Eggshell-Based Material in Electrochemistry

Although electrically non-conductive, ES and its membrane have been attracting great attention from electrochemists. Properties and applications of the ESM that are important from an electrochemical standpoint are summarized in a review paper (Baláž, 2014). Ever since, there have been growing numbers of papers dealing with this topic. To this end, herein we will describe recent progress concerning the utilization of both ES and ESM in the field of electrochemistry.

ESM in its unmodified form has only found its application as a separator in energy storage devices. Yu et al. demonstrated that an avian ESM separator could replace the conventional polypropylene separator in supercapacitors (Yu et al., 2012). Recently, Nguyen et al. studied eggshell membranes of various species (i.e., quail, chicken, goose) as separators in lithium-ion batteries (LIB) (Nguyen et al., 2018). It was established that different surface morphology, surface area and thickness of the ESMs affected the electrochemical performances such as discharging capacity, impedance and lithium-ion diffusion coefficient of Li-ion batteries.

On the other hand, ESM can be easily modified and consequently, high-performance electrode materials can be obtained. The presence of functional groups on the surface and high protein content make ESM suitable for various types of modifications. The functional groups have two important roles for the preparation of electrode materials: one is their ability to anchor electroactive metal precursors on ESM surface and the other is to reduce the metal cations to zero-valent metal nanoparticles. For instance, Selvacumari et al. showed that ESM can be used for the synthesis of metal oxides (i.e., SnO_2_) as a supercapacitor electrode material without using any toxic chemicals (Selvakumari et al., 2018). The suggested mechanism for SnO_2_ synthesis includes the reduction of the adsorbed Sn^2+^ on ESM into Sn^0^ by an aldehydic group naturally present in ESM. Upon annealing, SnO_2_ nanoparticles are formed through oxidation of Sn^0^. Meng and Deng demonstrated that NiO attached to carbonized ESM can be partially reduced to Ni in the reducing environment provided by released reducing agents from the ESM under heating (Meng and Deng, 2019). On the other hand, due to high protein content (rich in C and N), the ESM can be easily converted to functional carbon and heteroatom-doped (i.e., doped with nitrogen, oxygen and sulfur) carbon by its carbonization in an inert atmosphere. This results in an enormous potential of ESM application for energy conversion and storage devices (Li et al., 2012). Recent progress has been made in the development of composite materials based on non-noble metal compounds (oxides, sulfides etc.) and carbon derived from eggshell membrane. Meng and Deng used eggshell membrane as a bio-template for the formation of both carbon fibers and functional sulfides (Co_9_S_8_) based on *in situ* carbonization and sulfurization (Meng and Deng, 2016). This approach paved the way for the integrated use of eggshell waste and at the same time for the synthesis of functional nanostructures. A similar procedure was applied for syntheses of NiO (Lu et al., 2019), NiO-Ni (Meng and Deng, 2019), FeS (Zhao et al., 2019a), and Co_4_S_3_ (Xie et al., 2019) attached to the carbonized eggshell membrane (CESM) as electrodes. Improved electrochemical performance of composite materials compared to those of single component was attributed to the synergetic effect of CESM and metal oxide/sulfide nanoparticles. Moreover, Tong et al. demonstrated that FeCo alloy embedded in nitrogen self-doped carbon derived from ESM composite could be a good candidate for the substitution of commercial Pt/C (20%) electrocatalyst for oxygen reduction reaction (ORR) (Tong et al., 2017). The authors also found that pyrolysis temperature played a crucial role in controlling crystallinity, nitrogen and carbon content and textural properties of CESM and accordingly affected the electrocatalytic activity. Cui et al. also fabricated a competitive ORR electrocatalyst based on carbon nanofibers with a hierarchical structure, prepared by combining Co-containing zeolitic imidazolate frameworks with natural ESM (Cui et al., 2020). ESM was introduced to ensure the desired porous structure and to protect the aggregation of nanoparticles. As a result, they have noted the enhancement of mass transfer efficiency and consequently, electrocatalytic performance.

Recently, the research group of Pequendo de Olivera has recognized that eggshell membrane could be used for designing electrodes suitable for contemporary flexible supercapacitors (SCs) based on carbon nanostructures (CNs) material and conducting polymers (Alcaraz-Espinoza et al., 2017; da Silva et al., 2020). Fabrication of the supercapacitor electrodes often requires using binder materials or surfactants that could derogate electrochemical performance. On the contrary, authors suggested that flexible supercapacitor (SC) can be designed without the addition of auxiliary substances by the electrostatic assembly of CNs on pristine ESM (owing to the presence of functional groups), which was succeeded by the *in situ* polymerization of conducting polymer.

Utilization of ES has recently emerged in energy storage devices. Minakshi et al. have introduced the pioneering concept of using CaCO_3_ from eggshell as the cathode in Li-ion capacitors (LIC) in a non-aqueous electrolyte (Minakshi et al., 2018). Interestingly, the ES-based electrode showed a capacitance of 120 F g^−1^ (which was comparable to a classically activated carbon electrode under the same conditions) and good cyclic stability, with a capacity retention of 92% after 1,000 cycles. Moreover, the same group of authors concluded that eggshell-derived materials are suitable for the construction of electrode materials for aqueous energy storage devices (Minakshi et al., 2019). They built a symmetrical aqueous supercapacitor using chicken eggshell (CaCO_3_) as a cathode and its calcined form (CaO) as an anode, achieving the energy density of 14.5 W h kg^−1^ and power density of 525 W kg^−1^. In their latest work, Minakshi et al. fabricated a hybrid device comprising eggshell-derived CaO as a capacitor anode and NiO/Co_3_O_4_ as a pseudo-capacitor cathode with enhanced energy density up to 35 = W h kg^−1^ (Minakshi et al., 2020). Also, eggshell was utilized as an inexpensive Ca^2+^ (Foruzin et al., 2020) and CaO (Senthil et al., 2019) chemical source for the doping and coating of active electrode materials.

Recently, both ESM and ES have been investigated for electroanalytical purposes. ESM was used as a removable template for the synthesis of the Au/CeO_2_ 3D nanocomposite network (Liu et al., 2020a) and 3D Au porous network (Zhong et al., 2017) in the fabrication of electrochemical non-enyzmatic dopamine and glucose sensors, respectively. Superior electroanalytical performances of the developed sensors were attributed to the unblocked macroporous network and interwoven fiber structure of electroactive materials that provided small hindrance of analytes and great availability to active sites. Additionally, the rigid structure of the 3D network prevents the derogation of the sensor by aggregation and consequently improves operation and storage stability. On the other hand, it was shown that due to its porous structure and active nucleation sites ES can be employed as a support for nanoparticles and can be applied in the electroanalytical determination of various analytes. For instance, Au/CaCO_3_ was used for 4-nitrophenol electrochemical detection (Ding et al., 2020), while Fe_3_O_4_/eggshell composites were applied for the voltammetric determination of cadmium (Mohammadi et al., 2019).

In another report, Zhang et al. demonstrated a new approach for the utilization of every individual component of the egg in the formation of a 2D graphene-like carbon electrode and gel-like solid-state electrolyte (Zhang et al., 2019b). 2D graphene-like carbon was obtained by the carbonization of egg white/yolk at 650°C in argon with eggshell-derived CaCO_3_. Authors suggested that the CO_2_ released during the degradation of CaCO_3_ played a crucial role in the formation of 2D morphology with the high surface area of 593.1 m^2^g^−1^. Further activation of egg-derived carbon with KOH increased the surface area to 1,572 m^2^g^−1^. By mixing egg white/yolk with KOH, the gel-like solid electrolyte was obtained with competitive ionic conductivity and water preservation. Thereby, they introduced a concept that every part of the egg may be used for the construction of various parts of supercapacitor components. Fascinatingly, the authors constructed an all-solid flexible supercapacitor only based on the egg that exhibited the capacitance retention rate of 80% after 5,000 cycles at the current density of 1 A g^−1^.

### Limitations

Although excellent electrochemical performances of both ES and ESM were achieved, there are still some challenges that hamper their wide use. Most research in the field of electrochemistry has been focused on converting eggshell waste into a high-value carbon material and its further improvements by combining it with transition metals. On the other hand, it is unclear whether ESM-derived carbon can be fabricated in a reproducible manner. For instance, a small variation in the morphology of ESM-derived carbon may cause substantial changes in its electrochemical properties. Therefore, the lack of the reproducibility could present a major hurdle for large-scale application of ESM-derived carbon in electrochemical devices.

## Eggshell in Therapeutics

Both ES (mineral ES) and ESM (ES membrane) find various biomedical applications both as a raw natural source, and as components of synthesized materials. Numerous medical applications of ES (Rovensky et al., 2003; Baláž, 2018) and ESM (Baláž, 2014; Park et al., 2016; Sah and Rath, 2016) have been surveyed previously. Correspondingly, this review primarily discusses the advances which have been made since 2018.

### Biomedical Applications of Mineral Eggshell

ES is used as an inexpensive Ca^2+^ source for the development of medications (Rovensky et al., 2003; Siemiradzka et al., 2018), as particles for drug delivery (Jayasree et al., 2018; Verma et al., 2019), within organic matrix/mineral nanocomposites as potential tissue scaffolds in bone grafting (Apalangya et al., 2019; Shafiei et al., 2019; Trakoolwannachai et al., 2019; Wu et al., 2019; Ingole et al., 2020), and in food supplements (El-Shibiny et al., 2018; Islam et al., 2019; El-Zeftawy et al., 2020).

Calcium-containing medicines, food supplements, and vitamin-mineral complexes are mainly intended for the concomitant treatment of osteopenia and osteoporosis, and for the prevention of osteoporotic fractures. Osteoporosis is associated with a gradual decrease in bone mineral density (BMD) due to some imbalance in bone tissue remodeling when bone resorption, rather than bone formation prevails (Florencio-Silva et al., 2015). Bone resorption happens, for example, if the production of hormones and cytokines which trigger the process of osteogenesis subsides as a consequence of natural aging, immobilization, or from toxic effects of the environment. Resorption is initiated by osteoclasts. Within the lacunae formed by osteoclasts, osteoblasts create a new extracellular organic matrix (collagen type I), followed by a subsequent mineralization which yields precipitated crystals of the calcium-based mineral, hydroxyapatite (HA). Calcium also plays a crucial role in the transmission of intercellular and intracellular signals, and facilitates allosteric regulation in a series of biochemical reactions, thereby affecting both the differentiation and proliferation of cells, including bone-forming osteoblasts (Munaron, 2006; Blair et al., 2011). Therefore, bone resorption can be triggered by calcium-dependent PTH release and the subsequent osteoclast activation through the RANK/RANKL/OPG signaling pathway^1^ to compensate for the reduced concentration of calcium in the serum. So, the prevailing opinion is that calcium supplements can slow down the osteoporotic degradation of bone tissue.

Calcium is not, however, without its hazards. To avoid hypercalcemia and its negative health consequences, the calcium release profile of medicines and supplements should be provided properly, maintaining optimum calcium concentration in the blood serum. In a recent study (Siemiradzka et al., 2018), tablets were produced from the mineral ES, roasted at 120°C for 2 h, and sieved to unify grain size. Calcium citrate prepared using ES as raw material and calcium *bis*-glycinate were completely released within 150 min. At the same time, ES calcium carbonate added to calcium *bis*-glycinate prolonged the release of calcium ions to 4 h.

Evaluation of the optimum calcium release profile is also related closely to the determination of adequate dosages. Moreover, the range of effective dosages should be established for each indication. However, the biological response to the dose of administered calcium could be non-linear. The dosage dependence of ES calcium supplements on obese disorders in rats was studied for 26 weeks (El-Zeftawy et al., 2020). A low dose of ES supplement (7.2 g Ca^2+^ in form of ES/kg rat) for the treatment of rats with a high-fat diet led to significant enhancement of lipid profiles, liver enzymes, kidney functions, leptin, adiponectin, Ca^2+^, 25-hydroxyvitamin D, thyroid-stimulating hormone, free thyroxine, and PTH levels. The superoxide dismutase specific activity was elevated, thereby improving the antioxidant response. However, a high dose of Ca^2+^ (18 g Ca^2+^ in form of ES/kg rat) and a low-fat diet were less effective for the treatment of obese rats, as compared to providing rats with a low dose of ES alongside the high-fat diet. Thus, Ca^2+^ supplementation by ES can be regarded as a beneficial approach for obesity management with anti-cholesterol effects in low dose treatment. There was a reduction in weight gain, body weight, body mass index, blood glucose, insulin, and homeostasis model assessment of insulin resistance.

The main message of some studies is that the additional calcium sources should be included into the diet, to normalize the process of bone remodeling, because ≪a typical dietary calcium intake is not sufficient to satisfy the recommended daily calcium intake for all age groups≫ (El-Shibiny et al., 2018). To enhance the bioavailability of poorly soluble calcium salts, the eggshell powder is processed into the nano-sized state, which is characterized by high water absorption and lower zeta-potential as compared to micro ESP (eggshell powder) (El-Shibiny et al., 2018). ES was milled to achieve nano-sized particles (~10 nm) agglomerated into clusters of few μm. However, after ultrasound treatment of suspension and filtration through a 0.45 μm membrane filter, rapid aggregation of nano-sized particles was observed. This aggregation was attributed to the hydrophobic nature of the crystalline particles. Yogurt from the milk of buffalo and cows was fortified with nano-ESP up to 0.3% with no adverse effects or biochemical changes in the product.

The design of mineral composites themselves and organic matrix/mineral composites (OM/MC) as potential tissue scaffolds is another interesting application of ES. ESP or ESP-derived HA are implanted into the organic matrix, to enhance its mechanical properties and thermal stability, increasing simultaneously specific surface area to provide osteoblast-like cells infiltration and better adhesion (Apalangya et al., 2019; Trakoolwannachai et al., 2019; Wu et al., 2019). Ca-based minerals seem to stimulate osteoblast differentiation and proliferation *in vitro* and *in vivo*.

In a recent review (Opris et al., 2020) and original papers (Kattimani and Lingamaneni, 2019; Kattimani et al., 2019a,b) clinical studies on the effect of nano-dispersed HA as a substitute material in oral surgery were described in detail. Both ESP-derived HA and synthetic HA promoted bone regeneration displaying similar healing characteristics confirmed with histological analysis and microcomputed tomography.

OM/MC on the basis of ESP-reinforced microparticle hydrogels implanted in a rat model enabled the differentiation of pre-osteoblasts enhancing mineral deposition by these cells (Wu et al., 2019). The hydrogel porous matrix allowed the pre-osteoblast migration in 3D and provided integration of the ESP. Gelatin methacrylate-ESP composite did not generate inflammatory responses *in vivo* and integrated well with the host. More importantly, the osteogenic differentiation was induced in a concentration-dependent manner, without using a specialized osteogenic growth medium.

The shape of the particles and the associated specific surface area are important characteristics for cell viability in the process of bone grafting. A potential scaffold for tissue regeneration was described in Apalangya et al. (2019), where ESP-derived nano-dispersed HA and poly(lactic) acid electrospun fibers composite (PLA/HA) was fabricated. HA particles were obtained from a pre-milled ES/ethanol/water/propylene glycol suspension using two sets of 6 × 3 mm and 12 × 6 mm diameter stainless-steel balls simultaneously, followed by the addition of diammonium hydrogen phosphate and ammonium hydroxide solution. The resulting elongated milled particles ranged in lengths from 100 to 120 nm and their diameters ranged from 10 to 20 nm. The nanostructured HA agglomerates displayed a high specific surface area to enable a better biological activity of osteoblastic cells (Laranjeira et al., 2010). The porous OM/MC structure was suitable for cell infiltration and proper attachment.

The enhanced osteogenic activity was displayed in poly (ε-caprolactone) (PCL)/polyvinyl alcohol (PVA) nanofibrous scaffolds doped by both carbon dots and ES-derived calcium phosphates in Shafiei et al. (2019). Such doping demonstrated a synergetic effect, promoting a high osteogenic differentiation and proliferation rate.

Fiber/HA nanocomposites of another type were produced by synthesizing (2,2,6,6-tetramethylpiperidin-1-yl)oxyl (TEMPO)-oxidized cellulose nanofibrils (TCNFs), or cellulose nanocrystals (CNCs) with hydroxyapatite (HA) in varying composition ratios (Ingole et al., 2020). Ca(OH)_2_ obtained from eggshells and NH_4_H_2_PO_4_ used as HA precursors were mixed in a stoichiometric ratio of Ca/P = 1.67. The mixture was ground in an agate mortar with a pestle, and subsequently dissolved in distilled water and heated to form a HA solution. The solution was then ultrasonicated and subjected to uniaxial compression. HA with either nanocellulose type (nanofibrils or nanocrystals) was found to improve human osteoblast cell viabilities.

Chitosan was used as a natural organic matrix (as an alternative to synthetic matrices) of a prospective scaffold (Trakoolwannachai et al., 2019). Chitosan is a biomaterial produced by alkaline deacetylation of chitin, composed of b-(1-4)-D-glucosamine and β-(1-4)-N-acetyl-D-glucosamine (Santos et al., 2019). Among other benefits, chitosan demonstrates antioxidant, anti-allergic, anti-inflammatory, and antimicrobial activities (Santos et al., 2019). The last one is of utmost importance, to prevent typical adverse reactions during bone grafting and wound healing. 10–30 wt.% of hydroxyapatite was loaded into the chitosan film, binding to the hydrophilic –OH and NH_2_ and lowering the water content in the composite (Trakoolwannachai et al., 2019). To produce HA, orthophosphoric acid and ESP were co-precipitated with ammonium hydroxide, adjusting the pH of the solution to 10. The reinforcement of the matrix with HA particles imposes an obstacle for chitosan chain movement, thereby increasing the glass transition temperature while the melting point stays the same. The chitosan film with an increased roughness becomes a suitable support for cell growth.

ES-derived carbonated calcium deficient nano-HA can be used both in bone substitutes and in local drug delivery systems demonstrating the improved cellular response compared to synthetic calcium-deficient hydroxyapatite nanoparticles (Jayasree et al., 2018; Verma et al., 2019). Calcium hydroxide with residual carbonate content (ES heated at 805°C for 1 h) in distilled water suspension and diammonium hydrogen phosphate solution at Ca/P ratio of 1.61 were mixed and HA synthesis was accelerated by a microwave irradiation. Carbonate (${\text{CO}}_{3}^{2-}$) group substitution ~6% for the phosphate (${\text{PO}}_{4}^{3-}$) group in ES-derived HA reduced the crystallinity down to 4% compared to 15% in synthetic HA. The substitution suppressed crystal growth and increased the surface area and solubility. As a potential nano-carrier for the local drug delivery, ES-derived carbonated HA showed higher drug loading and releasing compared to the synthetic analog in the studies with doxycycline and curcumin.

#### Limitations

**Figure 5:**
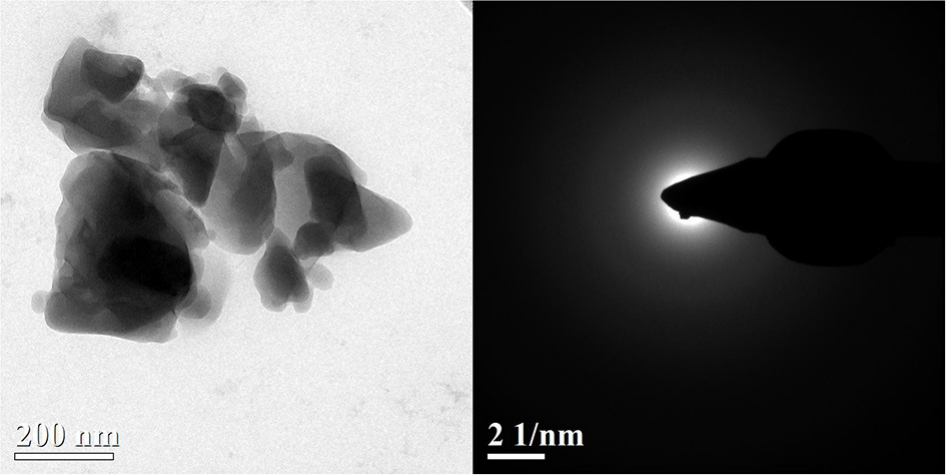
TEM of mechanically activated calcium salt of gluconic acid. Reprinted from Rybin et al. (2018) with permission from UdmFIC UB RAS.

In summary, *in vitro* and *in vivo* studies demonstrate low toxicity and substantial efficacy of ES-derived active pharmaceutical ingredients (APIs) for bone grafting. Nevertheless, discussions concerning the efficacy of calcium-containing supplements at osteoporosis treatment and preventing fractures continue. Numerous clinical studies and metadata analyses show equivocal results when using similar compounds (Rovensky et al., 2003; Grant et al., 2005; Reid et al., 2015; Tai et al., 2015; Yao et al., 2019; Reid and Bolland, 2020). Moreover, cardiovascular and gastrointestinal adverse events, together with renal calculi in calcium supplement use were reported to be likely (Reid et al., 2015; Reid and Bolland, 2020). The deficiency in regulative hormones and cytokines modulating calcium signaling pathways, such as cholecalciferol, PTH, and BMP2, disrupt proper calcium assimilation, as well as differentiation and proliferation of osteoblasts. That is why a mere intake of calcium supplements, without normalizing calcium metabolism, does not appear to resolve issues concerning a decrease in the magnitude of BMD. Instead, taking calcium supplements in this way can yield severe side-effects. In certain cases, some discrepancies in the conclusions of the published studies could be explained by the study design. It is important to know how the experimental protocol was controlled. Therefore, it is obvious that the choice of the correct experimental model for each indication when conducting preclinical studies is a priority task. Unfortunately, when developing the design of efficacy studies of ES-derived medicines, the physical state of a substance is rarely taken properly into account. At the same time, it is well-known that a drug should be considered as a material (Gardner et al., 2004; Boldyreva, 2016). The ability to deliver the medicine to targets substantially depends on the properties of API-containing compounds in the solid state, such as polymorphism, crystal shape, size, defects, etc. (Boldyreva, 2016). A striking example is the transformation of a crystalline API into an amorphous state, that can drastically modify both the pharmacokinetic and pharmacodynamic characteristics of a drug (Rams-Baron et al., 2018). For example, mechanical treatment of a complex based on calcium gluconate converts it into a nano-dispersed amorphous state (Figure 5) (Rybin et al., 2018). As a result, the physicochemical and biological properties of the complex change radically (Konygin et al., 2014; Rybin et al., 2014), converting it into a new non-protein regulator of calcium metabolism. Another example is amorphous calcium carbonate, which plays a significant role in biomineralization (Cartwright et al., 2012). The origin of the benefits of amorphous calcium carbonate on bone grafting and osteogenesis, as compared to the crystalline compound, still needs to be explored in detail. Thus, a comparative assessment of the clinical effectiveness between APIs manufactured from eggshells and from other sources should be carried out, considering the physical properties of drugs as materials, i.e., characterizing the samples by a complete set of structural parameters on microscopic, mesoscopic, and molecular scales.

### Biomedical Applications of Eggshell Membrane (ESM)

The eggshell membrane (ESM) is another important component, which is widely used for various applications, including in biomedicine, such as for the treatment of joint diseases, applications in reparative medicine (in particular, wound healing and sciatic nerve regeneration), and for producing vascular grafts (Park et al., 2016; Sah and Rath, 2016; Ruff et al., 2018; Hincke, 2019; Jalili-Firoozinezhad et al., 2020; Kulshreshtha et al., 2020).

ESM is a natural source of proteins, such as glucosamine and glycosaminoglycans, elastin, collagen (type I, V, X), hyaluronic acid, and other components that are related to the joint cartilage. The degradation of cartilage during osteoarthritis leads to bones rubbing, causing pain. ESM-based supplements were suggested to be used to slow down the progression of the disease. ESM suppresses the production of IL-1β and TNF-α inflammatory cytokines, subsiding inflammation (Hewlings et al., 2019). The double-blind and placebo study by Gil-Quintana et al. (2018) on the effect of daily intake of ESM supplement displayed short-term effects within the first 3 days. Joint pain was significantly reduced, as was joint dysfunction. In a randomized, double-blind, placebo-controlled clinical trial (Hewlings et al., 2019) the intake of water-soluble chicken eggshell membrane hydrolysate dietary supplement significantly reduced the joint stiffness in adults with knee osteoarthritis by the 5^th^ day of supplementation. One of the possible reasons for the development of osteoarthritis is the excessive presence of free radicals in the body. As osteoarthritis cannot be cured at the present time, early prevention of osteoarthritis is preferable to subsequent treatment. Antioxidant protection is considered to be amongst the most effective prevention methods. It has been shown that peptide fractions obtained by the enzymatic hydrolysis of ESM with Na_2_SO_3_ and alkaline protease combinations cope well with this task (Zhao et al., 2019b). After the separation, the identified water-soluble peptides with ESYHLPR and MFAEWQPR^2^ amino acid sequences exhibited the best antioxidant activities and could be used as highly efficient antioxidant agents.

Another interesting ESM application is its use in a wavy small-diameter vascular graft (Yan et al., 2020a,b). The internal membrane with the ESM surface modified with heparin and dopamine exhibited improved anticoagulation properties, simultaneously promoting human umbilical vein endothelial cell proliferation *in vitro*. The desired mechanical properties were provided by the formation of the composite with elastic thermoplastic polyurethane fibers.

Another important biomedical application of the ESM is wound healing (Balassa, 1971; Dawson, 2003; Sandri et al., 2013; Jun et al., 2014; Guarderas et al., 2016; Kiselioviene et al., 2016; Tummalapalli et al., 2016; Kuruoglu, 2017; Schmidt et al., 2017; Vuong et al., 2017, 2018; Kenny et al., 2018; Salah et al., 2018; Saporito et al., 2018; Augustine et al., 2019; Blaine and Thang, 2019; Huang et al., 2020; Kulshreshtha et al., 2020). Non-healing wounds are a major health problem worldwide and a significant cause of morbidity and mortality. Cutaneous wounds caused by burns, trauma, or other conditions, such as diabetic foot ulcers, can lead to serious infections, body fluid loss, as well as major medical burdens (Ahmed et al., 2019b). Effective treatments for acute and chronic skin wounds are the focus of intensive research. ESM is supposed to offer great solution also as a topical ingredient in skincare applications to maintain skin health by reducing bacterial infections and inflammation (Kulshreshtha et al., 2020).

ESM was used as an integral component of various formulations, to enhance wound healing, facilitate rapid endothelialization and anticoagulation (Yan et al., 2020a). It was suggested as an important component of various wound-care dressings (Guarderas et al., 2016; Kiselioviene et al., 2016; Li et al., 2016, 2019b,c; Krishnan et al., 2020). ESM was also used to produce hybrid nanofibrous scaffolds for cutaneous tissue engineering (Mohammadzadeh et al., 2019), materials for periodontal tissues (Motoji et al., 2020), and for sciatic nerve regeneration (Farjah et al., 2020). The nanofibrous scaffolds based on the natural extracellular matrix promote the dynamics of skin progenitor cells and accelerate differentiation into mature keratinocytes (Mohammadzadeh et al., 2019).

A wound dressing should act as a skin barrier during the wound healing process. Ideally, it should promote wound healing and prevent bacterial infection. It should exhibit good biocompatibility and appropriate porosity, as well as effective antibacterial activity (Liu et al., 2020b). Only few clinical products can meet all these needs due to their mono-functionality, relatively complicated preparation procedures, and high cost. Developing such an ideal wound dressing for tissue regeneration remains a significant challenge. Natural materials are often multifunctional, owing to their complex composition and structure; ESM is no exception in this respect. Its successful use in various capacities for wound healing has been documented in numerous original research papers, reviews, book chapters, and books, as well as patents (Balassa, 1971; Dawson, 2003; Sandri et al., 2013; Jun et al., 2014; Guarderas et al., 2016; Kiselioviene et al., 2016; Tummalapalli et al., 2016; Kuruoglu, 2017; Schmidt et al., 2017; Vuong et al., 2017, 2018; Kenny et al., 2018; Salah et al., 2018; Saporito et al., 2018; Augustine et al., 2019; Blaine and Thang, 2019; Huang et al., 2020; Kulshreshtha et al., 2020). Still, the mechanisms of action of the ESM and its role in the already successfully tested materials for wound healing remains hardly explored. Most likely, there is no single mechanism, and depending on the formulation and the application, the ESM can be either an active ingredient or an excipient.

ESM contains an abundance of antimicrobial, immunomodulatory, and other bioactive proteins, peptides, amino acids, collagen-like proteins, enzymes, and glycosaminoglycans (Yamauchi et al., 2013; Yoo et al., 2014; Benson et al., 2016; Jensen et al., 2016; Ahmed et al., 2017, 2019a,b; Niu et al., 2017; Dubourdieu, 2019; Gautron et al., 2019; Zhao et al., 2019b; Zhu, 2020). Each of them can have a pronounced influence on wound healing as a biologically active compound. Proteoglycans in ESM have been used successfully in treating non-healing wounds and burns, due to their biocompatibility, biodegradability, and similarity to macromolecules found in the human body (Benson et al., 2016; Mohammadzadeh et al., 2019).

Collagen (several types, in particular I, V, X) was named as one of the components that accounts for the biological activity of ESM (Yamauchi et al., 2013; Benson et al., 2016; Tummalapalli et al., 2016; Ahmed et al., 2017, 2019a,b; Dubourdieu, 2019; Mohammadzadeh et al., 2019; Farjah et al., 2020; Liu et al., 2020b; Motoji et al., 2020). ESM exerts an anti-aging effect by increasing type III collagen levels (Motoji et al., 2020). ESM has been shown to upregulate the expression of both collagen types I and III (Yamauchi et al., 2013).

Proteomic analysis of processed eggshell membrane powder (PEP) identified 110 proteins, including structural proteins such as collagen and cysteine-rich eggshell membrane proteins (CREMPs) that together constitute about 40% of PEP. Functional annotation clustering showed various predicted functionalities related to wound healing, including response to an external stimulus, defense response, inflammatory response, and cell-substrate adhesion (Ahmed et al., 2017, 2019a,b).

The presence of essential structural proteins such as osteopontin, sialoprotein, keratin, proteoglycans, and glycoproteins has been previously proved (Wong et al., 1984; Chowdhury, 1990; Arias et al., 1991; Nakano et al., 2003; Zhao and Chi, 2009; Hincke et al., 2012; Dombre et al., 2017; Silva et al., 2018). Also, ESM harbors numerous natural glycoproteins, notably glucosamine, chondroitin, and hyaluronic acid, which are applicable for the cutaneous wound dressings (Mohammadzadeh et al., 2019).

The ESM enzymatic hydrolysate possesses a remarkable antibacterial activity (Yoo et al., 2014; Niu et al., 2017). A hydrolyzed water-soluble ESM product triggered upregulation of antioxidant-response elements in human keratinocytes, and significantly reduced production of reactive oxygen species by polymorphonuclear (PMN) cells *in vitro*. Furthermore, human dermal fibroblasts treated with soluble ESM *in vitro* showed an increase in the production and secretion of collagen and elastin (Benson et al., 2016; Augustine et al., 2019). Usually, the enzymatic hydrolysis is used to obtain solubilized proteins because both acid and basic hydrolysis destroy some constituents, decreasing the nutritional value of proteins (García and González, 2020). Solubilization of ESM, however, requires a more complicated approach due to the high resistance of ESM to the enzymes. In the study by García and González (2020), denaturing agent with detergent properties (sodium lauryl sulfate or taurocholate)/reducing agent (dithiothreitol and sodium metabisulfite) compositions were patented to break disulfide bridges of ESM proteins and provide access to the enzymes (cysteine proteases), facilitating the hydrolysis.

In many studies the ESM is used as a template or framework, forming a composite with bioactive molecules or nanoparticles embedded into its structure (Jun et al., 2014; Li et al., 2016, 2019b; Puertas-Bartolome et al., 2018; Augustine et al., 2019; Raz et al., 2019; Farjah et al., 2020; Krishnan et al., 2020; Selvam et al., 2020). Consisting of unique interwoven shell membrane fibers, the ESM provides a unique supporting platform for functional nanoparticles in catalysis and sensing (Vuong et al., 2017). The same or similar materials can be used for wound healing. The flexible and highly pure microfibrous network structure of the ESM can be used as an artificial extracellular matrix (ECM) platform for engraftment or as a tissue-engineered scaffold (Vuong et al., 2017, 2018; Park et al., 2019; Liu et al., 2020b).

Flexible and functional scaffolds were constructed, for example using an ESM and graphene. The graphene-layered ESM (GEM) scaffolds showed better mechanical and hydrophilic properties than those of a raw ESM. The GEM scaffolds can control the adhesion properties of stem cells, enhancing the proliferation and osteogenic properties of the cells as compared to the effects of a raw ESM. Additionally, the GEM scaffolds can improve the secretion of growth factors from stem cells, possibly through enhanced cell–substrate interactions, thereby promoting the proliferation and differentiation of these cells (Park et al., 2019). Physical and biochemical features of a collagen membrane can be significantly improved by conjugating it with soluble ESM proteins (Ino et al., 2006).

Some studies emphasize the positive effect of lycopene on ESM guidance channels in sciatic nerve regeneration in rats, with ESM acting as a nerve guidance channel (NGC). Ideally, NGC must be biodegradable, biocompatible, flexible, semipermeable, easily made and sterilized, and be amenable to long-term storage. A composite of the ESM with lycopene meets all of these requirements. There are many benefits of using an ESM channel, including low cost and flexibility. Moreover, ESM is strong enough to maintain a suture. In addition, the dimensions of the channel are easily controlled. At the same time, the ESM is not merely a “guiding structure.” As was mentioned above, it contains several types of collagen, hyaluronic acid, and laminin. These biologically active compounds are important in nerve regeneration (Farjah et al., 2020).

Another example of using the ESM as a framework for a biologically active composite is to use it as a porogen of the chitosan-based macroparticles, which are used to immobilize protease. Chitosan-based macroparticles are a common carrier for enzyme immobilization that is applied in the food industry. Driven by the requirement of large carrier pores for the biomacromolecular substrates, such as a protein, the eggshell membrane powder (ESMP) was employed as a multifunctional porogen to improve the physicochemical structure of chitosan-based macroparticles. The results showed that an increase of ESMP percentage could improve the porosity of macroholes in macroparticles, and it also enlarged the size of mesopores. Moreover, the ESMP significantly increased the amount of papain immobilization, whereas the specific activity of immobilized papain went through a maximum with the increase of ESMP. The inclusion of 20% ESMP in chitosan-based macroparticles gave the highest activity of its immobilized protease (Liu et al., 2020b). The ESM-chitosan blend films were also used as wound-care dressings (Li et al., 2019c).

Composites of ESM with nanoparticles of biologically active compounds, such as metal oxides or metals, were successfully applied for wound healing. CdO/ZnO-ESM nanocomposites were shown to have an exceptional antimicrobial activity against both Gram-positive and Gram-negative bacteria (Selvam et al., 2020). A copper-containing bioactive glass (Cu-BG) nanocoating (40–50 nm) with a uniform nanostructure was formed on a natural eggshell membrane (Cu-BG/ESM) by pulsed laser deposition (PLD). It was characterized by improved angiogenesis, antibacterial activity, and wound healing. The surface physicochemical properties, including the hydrophilicity and the hardness of ESM, were significantly improved after depositing Cu-BG nanocoatings. The 5Cu-BG/ESM films (containing 5 mol% Cu) could maintain a sustained release of Cu^2+^ ions and distinctly inhibit the viability of bacteria (Escherichia coli) (Li et al., 2016).

An antibacterial nanobiomaterial for wound-care based on the absorption of silver nanoparticles (Ag NPs) on the ESM was proposed in Raz et al. (2019), Li et al. (2019b), and Krishnan et al. (2020). The addition of Ag NPs changed the ESM from hydrophobic to hydrophilic, which is important for the wound-healing process. The Ag NPs/ESM composites had a higher surface area (159.08 m^2^/g), than the natural ESM (24.32 m^2^/g) and a suitable average pore size (10.92 nm). Hence, Ag NPs/ESM composites displayed better absorption and antibacterial abilities (Krishnan et al., 2020). Several techniques to produce composite materials based on ESM and Ag NPs have been reported. One of them includes pre-treating the eggshell membrane with procyanidin to reduce silver ions into nanoparticles, which are then incorporated into the membrane structure. Such materials showed antibacterial activity against *S. aureus, S. albus*, and *E. coli* when tested on bacterial plates (Krishnan et al., 2020).

Some ESM-based antibacterial materials for wound healing contain both metal and metal oxide nanoparticles. A challenge in fabricating such materials is to produce an effective carrier or delivery matrix to achieve a sustained release profile with high bactericidal efficacy along with good cytocompatibility. A facile route has been proposed to fabricate a hierarchical nanobiocomposite with effective loading of ZnO/silver nanoparticles (Ag NPs) to attain excellent bactericidal efficacy with a good and sustainable release profile. In the mentioned study, surface-functionalized eggshell membranes (ESM) were deployed as three-dimensional loading matrices for efficient loading of ZnO/Ag NPs (Raz et al., 2019). A simple sonochemically guided approach was adopted to synthesize ZnO nanoflakes *in situ* onto the microfibrous ESM and decorate them with Ag NPs, thereby forming a nanobiocomposite. The microstructural analysis confirmed the successful anchorage of ZnO nanoflakes and Ag NPs on microfibrous eggshell membrane, thus reinstating the hierarchical morphology of the nanobiocomposites. Owing to the synergistic activity of ZnO and Ag NPs, the nanobiocomposites demonstrated exceptional bactericidal activity against Gram-negative, *E. coli* or *P. aeruginosa*, and Gram-positive, *S. aureus* or *B. subtilis*, bacterial cells. Furthermore, direct exposure of nanobiocomposites with NIH 3T3 cells revealed the biocompatible nature of developed matrices. Prolonged exposure also indicated that the 3T3 cells tend to adhere onto the microfibrous nanobiocomposite without any observable deformation in cellular morphology. The architectural tribology and excellent bactericidal performance of the nanobiocomposites along with their cytocompatible nature manifested its potential as an alternative platform for various biomedical applications.

There are also examples when ESM is embedded into another matrix. For example, ethanol-lubricated expanded-polytetrafluoroethylene vascular grafts loaded with an eggshell membrane extract and heparin were applied for rapid endothelialization and anticoagulation (Yan et al., 2020b). This novel ethanol-water lubricant not only evenly dispersed the ESM extract but also dissolved the heparin sodium. The as-fabricated synthetic expanded polytetrafluorethylene (ePTFE) grafts, which are routinely used for vascular repair and reconstruction, showed the classic node-fiber structure suitable for cell adhesion and migration. The embedded ESM extract and heparin improved the hydrophilicity and cytocompatibility, resulting in enhanced cell viability and proliferation of human umbilical vein endothelial cells (HUVECs) (Yan et al., 2020b).

Chitosan films enriched with the ESM powder were produced in Santos et al. (2019), Li et al. (2019c), and Liu et al. (2020b). Their thermal, chemical, biological, and mechanical properties were studied and the films were shown to be suitable for biomedical applications (Santos et al., 2019).

An example of a complex composite containing ESM is provided by a biodegradable dual porous PLA-PVA core-shell fiber, enriched with ESM, into which a connective tissue growth factor (CTGF) is also incorporated (Augustine et al., 2019). CTGF is a signaling molecule with several roles in tissue repair and regeneration including promoting cell adhesion, cell migration, cell proliferation, and angiogenesis. The incorporation of CTGF into a fiber facilitates its sustained release. The membranes were fabricated by a core-shell electrospinning technique. CTGF was entrapped within the PVA core which was coated by a thin layer of PLA. This biomaterial can be used to treat diabetic wounds (Augustine et al., 2019).

As another example, one can mention the nanofibrous scaffolds composed of a blend of poly(ε-caprolactone) (PCL), silk fibroin (SF), soluble eggshell membrane (SESM), and Aloe vera (AV) gel (Mohammadzadeh et al., 2019). Such composites were also developed by electrospinning. These scaffolds were applied for cutaneous tissue engineering. The artificial scaffolds were modulated with natural biopolymers, including the ESM as one of them. In the natural ESM, cross-linked disulfide bonds limit the solubility of ESM and its use in biomedical engineering as the nanofibrous scaffold. Soluble ESM (SESM) can be prepared, but it has poor mechanical properties because of its low molecular weight and wide molecular weight dispersion. Therefore, it is difficult to obtain a nanofibre or film from pure ESM. Adding poly(ε-caprolactone) (PCL) to the composite materials improves the mechanical properties, without deterioration of biocompatibility (Mohammadzadeh et al., 2019).

#### Limitations

Summing up, one can note that most publications on the biomedical applications of the ES and ESM either report the preparative techniques of producing a material, or document the results of its *in vitro* and *in vivo* testing. Despite many impressive examples of successful applications of these materials, little remains known about the underlying mechanisms of action. This field looks like a *terra incognita* for solid-state physical chemists and can promise many exciting fundamental discoveries, if biochemical, medical, and solid-state research is carried out jointly by interdisciplinary teams.

One of the technological challenges when producing an ESM-based material is to separate ESM from the shell. The poor solubility of ESM limits the bioavailability of its constituents and reduces the expression of their potential bioactivity. This problem can be overcome, for example by cryo-grinding and homogenization. This reduces the particle size and produces a particalized eggshell membrane (PEM) approaching submicron dimensions, with enhanced anti-inflammatory and antimicrobial activity against skin-associated pathogens (Kulshreshtha et al., 2020).

## Using Tools of Mechanochemistry to Boost the Application Potential of Eggshell Waste: an Update

**Figure 6:**
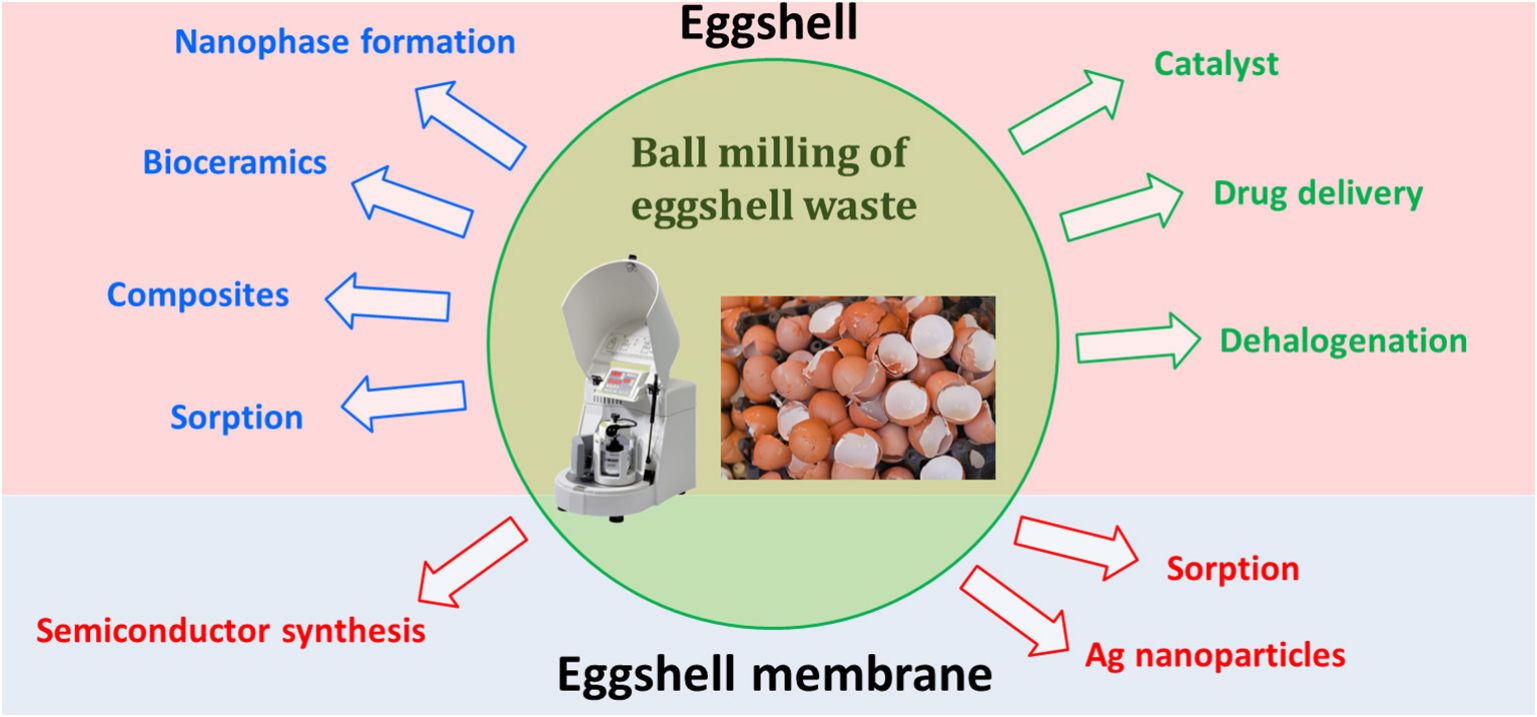
Various applications of eggshell and its membrane subjected to mechanochemical treatment. Reprinted from Baláž (2018), Copyright (2018), with permission from Elsevier.

Mechanochemistry is already an established method in various research fields and is gaining more and more interest in the research world (Boldyrev and Avvakumov, 1971; Butyagin, 1971, 1994; Heinicke, 1984; Tkáčová, 1989; Gutman, 1998; Avvakumov et al., 2001; Boldyrev, 2006, 2018; Baláž, 2008; Zyryanov, 2008; Declerck et al., 2009; Guo et al., 2010; James et al., 2012; Baláž et al., 2013; Boldyreva, 2013; Braga et al., 2013; Groote et al., 2013; Huot et al., 2013; Šepelák et al., 2013; Stolle and Ranu, 2014; Cagnetta et al., 2016; Andre et al., 2017; Colacino et al., 2018; Bolm and Hernandez, 2019; Bychkov et al., 2019; Gomollón-Bel, 2019; Rogachev, 2019; Suryanarayana, 2019; Tan and García, 2019; De Oliveira et al., 2020a,b), has recently been awarded on a European level by a COST project (Baláž et al., 2019b; Hernández et al., 2020; www.mechsustind.eu)^3^ and is also recognized by IUPAC (Mcnaught and Wilkinson, 1997; Gomollón-Bel, 2019). International Mechanochemical Association is IUPAC-affiliated since 1988 (www.imamechanochemical.com/about-us/)^4^. The area of waste treatment is also interesting for the mechanochemical community (Lomovsky and Boldyrev, 2006; Guo et al., 2010; Tan and Li, 2015; Cagnetta et al., 2016; Bychkov et al., 2019; Li et al., 2019a; Piras et al., 2019). There are several papers that applied tools of mechanochemistry for the treatment of eggshell waste. A review paper on this topic was published in 2018 (Baláž, 2018). Ball milling was applied for four main purposes- formation of nanophase, synthesis of bioceramics, composite materials synthesis, and improvement in sorption properties. The broad application fields upon application of ball milling of eggshell waste can be well-seen from the graphical abstract of that paper (Figure 6).

Since the publication of the review article mentioned above, a couple of new works have appeared. The most important ones with their main findings are briefly reviewed in Table 1. As follows from the table, the authors targeted different application fields, however, the majority of them fall into the categories outlined in Figure 6. The works on bioceramics preparation and the preparation of nano-eggshell dominate. Nevertheless, some new application spheres have emerged, e.g., biogas production (Sari et al., 2020), filtration (Seeharaj et al., 2019), dentistry (Onwubu et al., 2019a,b), and electrochemistry (Cherdchom et al., 2019; Senthil et al., 2019; Karuppiah et al., 2020). A reference to the study using ball milling of eggshell for the application in catalysis (Mosaddegh and Hassankhani, 2014) has been already mentioned earlier. A few interesting reports are summarized in more detail below.

**Table 1:** Overview of research studies applying a mechanochemical approach for the treatment of eggshell waste published recently: experimental techniques, the most important result and application field of the final product.

**Milling input**	**Experimental techniques**	**Most important results**	**Final product/application**	**References**
Eggshell	SEM	Statistical approach; milling speed is the most important factor	Hydroxyapatite/bioceramics	(van Hoten et al., 2018)
Eggshell + ethanol/water	XRD, SEM, TEM, TA, optical microscopy, mechanical properties evaluation, biocompatibility	5 wt% nanohydroxyapatite was the ebst, cells grow on the fibers	PLA-nanohydroxyapatite fibers/bioengineering	(Apalangya et al., 2019)
Eggshell + window parapet made of PVC	XRD, FTIR, titration	Comparison of planetary and vibratory milling, scalability	Calcium chloride + harmless organic matrix/dechlorination	(Baláž et al., 2019a)
Eggshell + TiO_2_/+ Mg	XRD, TA, SEM	Comparison of conventional and high-energy ball milling	CaTiO_3_ ceramics/electronics	(Cherdchom et al., 2019)
Eggshell/cuttlefish bone+ phosphoric acid	SEM, XRD, Raman, TEM	No sintering, comparison of eggshell and cuttlefish bone as Ca sources	Hydroxyapatite/bioceramics	(Ferro and Guedes, 2019)
Eggshell + rice straw	XRD, SEM, adsorption kinetics and thermodynamics and influence of various factors	Maximum sorption capacity of 231 mg/g was evidenced for balanced eggshell:rice straw ratio	Phosphate ions adsorbent/wastewater treatment	(Liu et al., 2019)
Eggshell + acetone	XRD, SEM	Sintering enriched Ca content and did not result in a significant increase in crystallite size	Nanoization	(Puspitasari et al., 2019)
CaO from eggshell	XRD, FTIR, SEM, fluorescent microscopy, biocompatibility evaluation	Comparison of ball milling, mortar and pestle and Food and Drug Administration (FDA)-approved methodology, post-milling reaction with H_3_PO_4_	β-tricalcium phosphate scaffolds/bioceramics	(Roopavath et al., 2019)
CaO from eggshell	XRD, SEM, chemical oxygen demand, biogas and methane production	Size reduction into nano-range resulted in a significant improvement in biogas production	Biogas production from palm oil mill effluent: cow manure mixture	(Sari et al., 2020)
Eggshell + ethanol	XRD, FTIR, SEM, WCA, SPM	Stearic acid favors the transformation into aragonite	Superhydrophobic eggshell/filtration	(Seeharaj et al., 2019)
Eggshell + Li-Ni_0.8_Co_0.1_Mn_0.1_O_2_	TA, XRD, FTIR, SEM/EDS, XPS, electrochemical measurements	CaO prevents electrolyte dissolution and electrode corrosion	CaO-coated Li-Ni_0.8_Co_0.1_Mn_0.1_O_2_ electrode/electrochemistry	(Senthil et al., 2019)
Eggshell + acetone	XRD, SEM, FTIR	Comparison of calcined (CaO) and non/calcined (CaCO_3_) material	Nanoization	(Supriyanto et al., 2019)
Eggshell + stearic acid/water	XRD, TA, TEM	Stearic acid reduces the crystallite size and thermal degradation temperature	Nanoization	(Villarreal-Lucio et al., 2019)
Eggshell + aqueous solution of phosphate precursor	XRD, FTIR, SEM, TA	Pure HA produced from different precursors using three different CaCO_3_ sources using wet milling and low-temperature treatment	Hydroxyapatite/bioceramics	(Cestari et al., 2020)
Eggshell + ethanol	S_BET_, particle size distribution, zeta potential, SEM, TEM, EDS, FTIR, Ca^2+^ concentration determination, XRD	Zeta potential was decreased during treatment	Nanoization	(Huang et al., 2020)
Eggshell membrane + Li_2_FeSiO_4_	XRD, TA, S_BET_, Raman, XPS, TEM	ESM served as a carbon source for improving electrical properties of the LFS ESM composite	LFS-C composite/electrochemistry	(Karuppiah et al., 2020)
Eggshell + Al_2_O_3_	SEM, mechanical properties, corrosion, thermal expansion	Toughness and ductility reduced, but tensile strength, hardness, corrosion resistance, thermal stability improved upon addition of CaO derived from eggshell	Al/eggshell/Al_2_O_3_ composite	(Kumar, S., Dwivedi, S. P., and Dwivedi et al., 2020)
Eggshell	Particle size distribution, SEM, EDX, XRD	The authors report graphite in the eggshell	Micronization	(Ononiwu and Akinlabi, 2020)
Eggshell/eggshell + TiO_2_	FTIR, TEM, XRD, acid-resistant and buffering properties	The buffering performance was evaluated against that of four available toothpastes	Eggshell-TiO_2_ composite/dentistry	(Onwubu et al., 2019a)
Eggshell/eggshell + TiO_2_	The same as above, but also SEM	The tooth surface is less destroyed when using Colgate toothpaste and the prepared composite in comparison with other toothpastes	Eggshell-TiO_2_ composite/dentistry	(Onwubu et al., 2019b)
Eggshell	XRD, SEM, TEM, FTIR, mechanical properties, microhardness, erosion resistance	Different amounts of eggshell (from 1-4%) in the composites were beneficial for different mechanical properties	Eggshell-epoxy composite/composites	(Panchal et al., 2020)
Eggshell + acetone	XRD, FTIR, Raman, SEM	Effect of various post-milling sintering temperatures (900–1,200°C) on CaCO_3_-CaO transformation was investigated	Nanoization	(Puspitasari et al., 2020)
Eggshell	Particle size, SEM, XRD, AFM, mechanical properties, chloride ion permeability	Improvement of mechanical properties of oil well cement and accelerate hydration process	Oil well cement-eggshell composite	(Salman et al., 2020)

**Figure 7:**
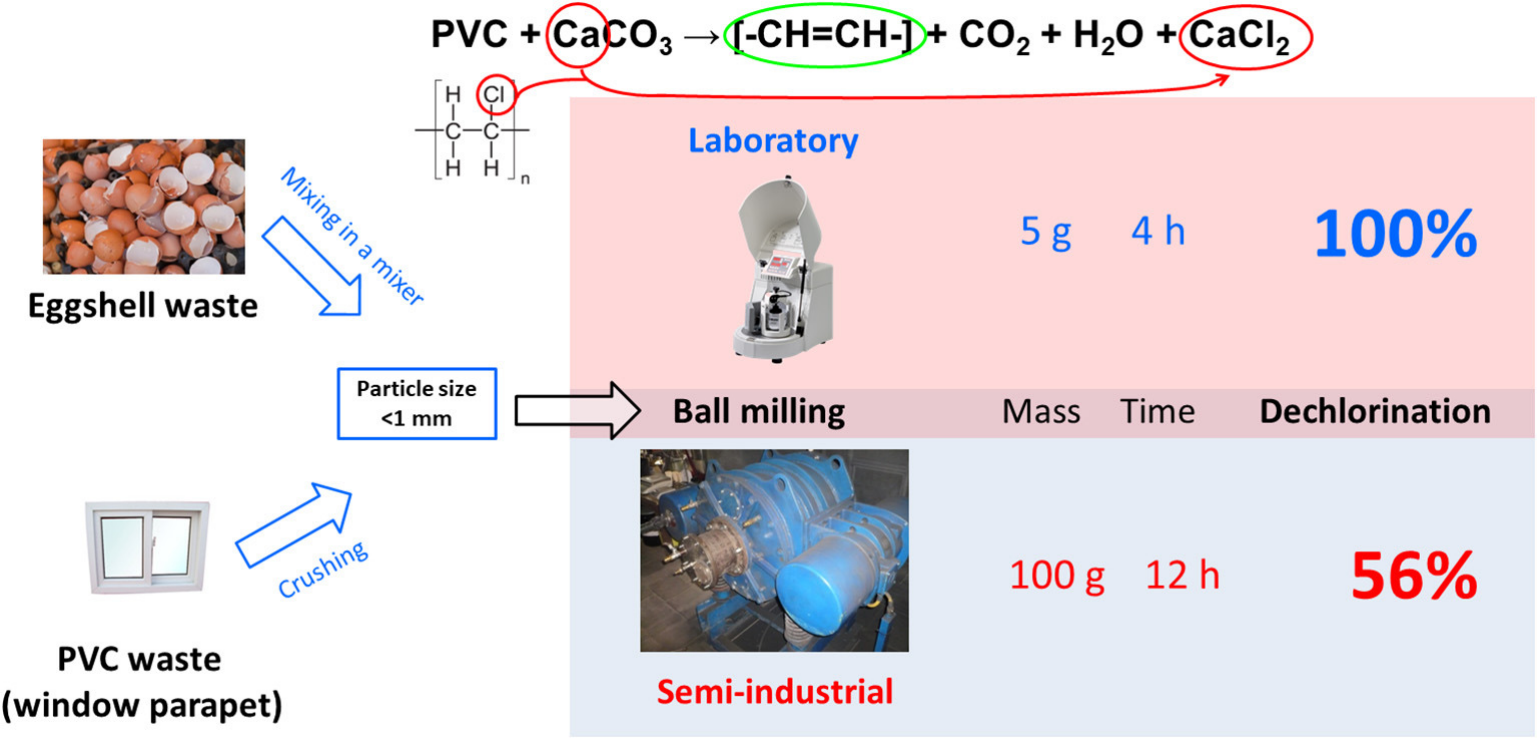
Schematic illustration of main idea and results of the laboratory and semi-industrial dechlorination of PVC waste using eggshell. Reprinted from Baláž et al. (2019a), Copyright (2019), with permission from Elsevier.

The idea of dechlorinating PVC waste using eggshell (containing both shell and membranes) was scaled up to a semi-industrial level and compared to a lab-scale process in Baláž et al. (2019a). The laboratory-scale experiment was more efficient, as complete dechlorination was observed upon 4 h of milling, whereas only 56% was reached after 12 h of treatment in a semi-industrial vibratory ball mill. However, the milling conditions in the vibratory mill were not optimized. The rate constant obtained using the best-fitting zero-order kinetic model for the scaled-up processing was also significantly lower. Chlorine originating from waste PVC was successfully bound to a deliquescent CaCl_2_. The idea, scale, and dechlorination result can be well-seen in Figure 7.

From the papers focusing on the bioceramics production, the report (Ferro and Guedes, 2019) is of particular interest, as the authors succeeded in the preparation of hydroxyapatite just by using ball milling without the need of subsequent sintering. As calcium sources either eggshell or cuttlefish bones (containing Ca in the form of calcite or aragonite, respectively), have been applied. The reaction progress was found to be a function of energy input and was different for each precursor. The final product was formed easier when using an aragonite-containing precursor.

Eggshell was co-milled with rice straw and subsequently heated at 800°C in order to obtain effective adsorbent of phosphate ions in Liu et al. (2019). The kinetics of the adsorption process can be well-described by a pseudo-second-order kinetics and the maximum adsorption capacity calculated from the adsorption isotherm using Langmuir model was 231 mg/g (evidenced for eggshell: rice straw 1:1 ratio). The adsorption ability of all three phosphate ions (including the hydrogenated ones) has been investigated, yielding the best results for hydrogen-free ${\text{PO}}_{4}^{3-}$ ones, as the presence of hydrogen hampers the efficient complexation of Ca and P. The elevated temperature improved the outcome, which, together with other results in the paper confirmed the chemical character of adsorption.

**Figure 8:**
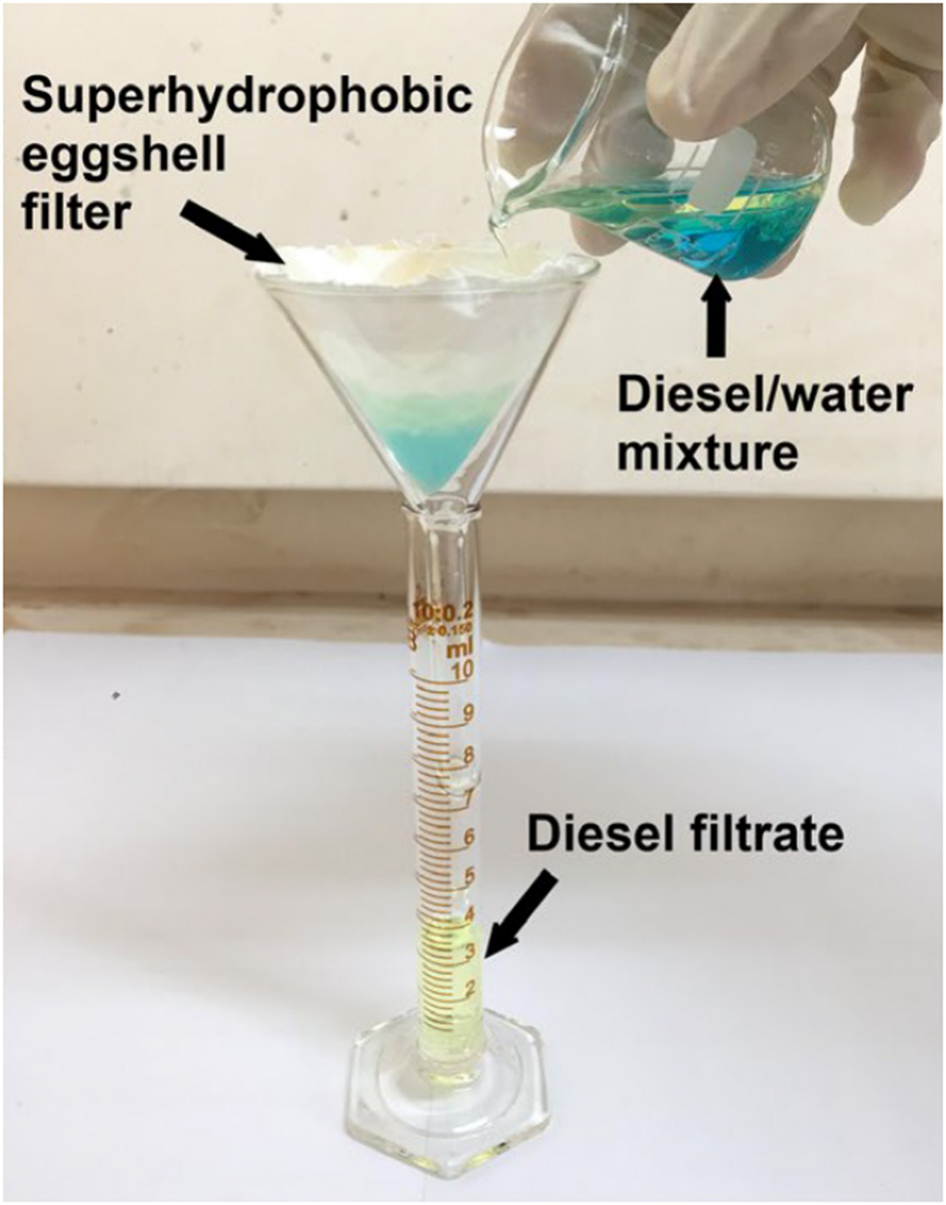
A photograph of the experimental setup for diesel/water separation test (water was dyed blue with methylene blue for better contrast) (Seeharaj et al., 2019) copyright 2019. The American Ceramic Society.

The hydrophobic eggshell waste was prepared by mechanical activation and subsequent modification with stearic acid mixed with polymer binder in Seeharaj et al. (2019). The obtained material was then dip-coated on glass and cotton fabric. As the resulting material exhibited the water contact angle values above 150°, it can be considered superhydrophobic. As a proof of the applicability, the separation of diesel/water mixture by filtration on the prepared material is shown in Figure 8. Whereas, diesel easily goes through to the filtrate, water remains on the filter.

Three different biogenic sources, namely eggshell (ES), cuttlefish bone (CB) and mussel shells (MS) have been used as sources of calcium to produce hydroxyapatite in Cestari et al. (2020). As a source of phosphates, mainly ammonium phosphate dibasic (APD) has been used. The XRD results showed that ES and MS contain mostly calcite and CB contains aragonite. On the contrary to Ferro and Guedes (2019), just milling did not yield the desired phases, however, a subsequent low-temperature treatment did. The temperatures in the range 120–150°C were sufficient to get the hydroxyapatite and the process seems to be the most straightforward (no intermediate phases) for the aragonite-containing CB precursors. Calcite phase could still be detected when using ES and MS, whereas pure HA was formed in the case of CB. Nevertheless, performing the reaction at low pH using phosphoric acid instead of APD yielded phase-pure hydroxyapatite also in the case of ES and MS precursors.

An interesting study showing the acid-resistant properties of the eggshell waste, which can be potentially applied in dentistry has been reported in Onwubu et al. (2019b). Enamels collected from bovine were exposed to the action of HCl in the presence of various toothpaste solutions and also to pure eggshell and eggshell-TiO_2_ composite. The acid-resistant properties could be well-traced from the SEM images of the enamels taken after the action of the corresponding solutions. Whereas, the enamels after the action of Colgate toothpaste, eggshell and eggshell/TiO_2_ is smooth, it contains fractures and roughness, being the result of acid action in the other cases.

**Figure 9:**
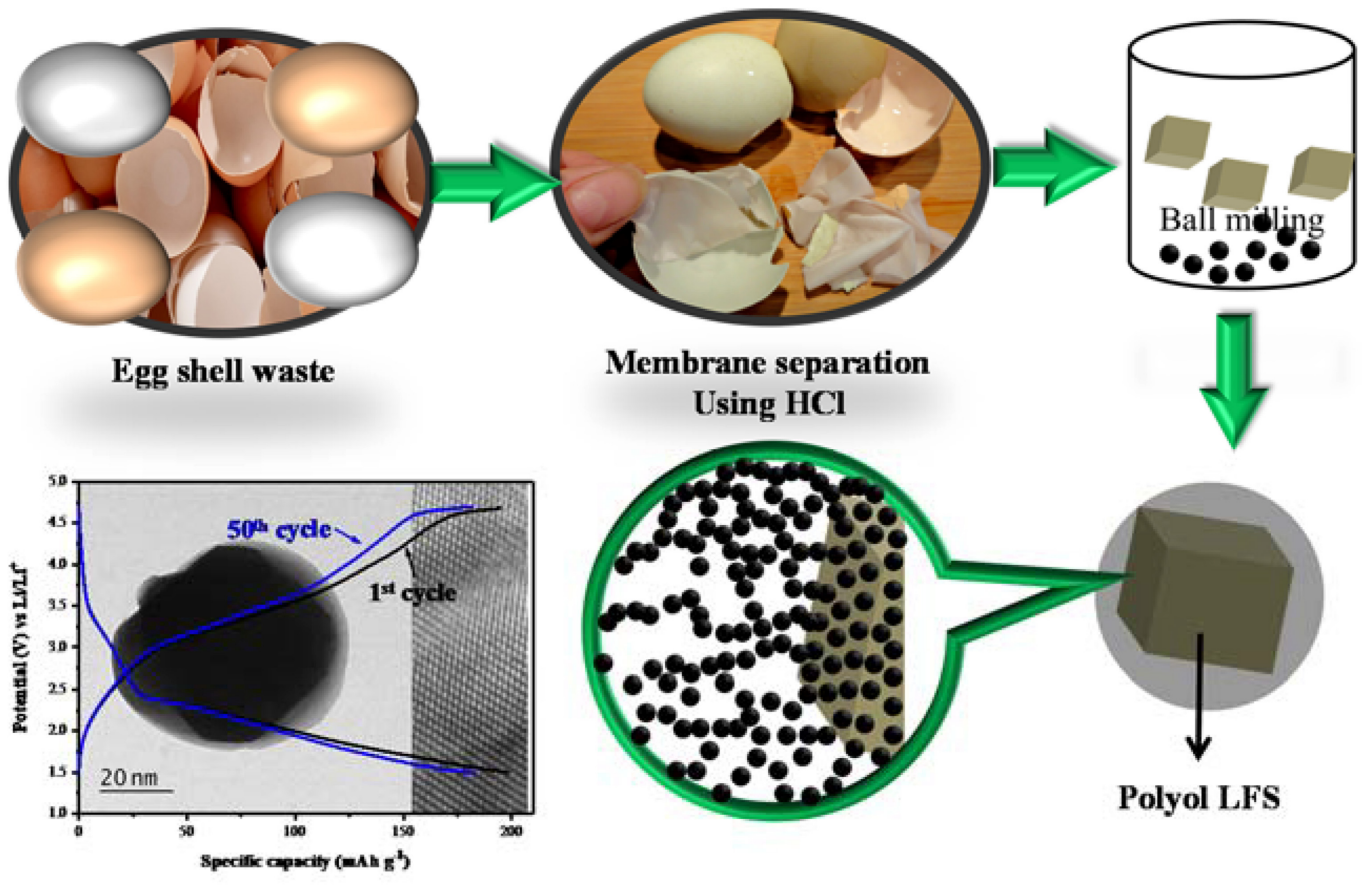
Schematic illustration of the procedure leading to the production of Li_2_FeSiO_4_/C composite with good electrical properties (Karuppiah et al., 2020).

Lithium iron orthosilicate Li_2_FeSiO_4_ (LFS) was prepared using the polyol method and subsequently co-milled with eggshell membrane serving as a carbon source in Karuppiah et al. (2020). The complete carbonization of ESM was achieved during the post-milling calcination in Ar atmosphere and the successful coating with iron was proven by TEM. The as-received LFS/C composite exhibited excellent electrical properties with high coulombic efficiency of 98.5% which can be maintained for more cycles. The main message of the paper is demonstrated in Figure 9.

The calcined eggshell was used to produce biogas from palm oil mill effluent and cow manure mixture in Sari et al. (2020). Namely, the effect of the particle size reduction achieved by ball milling was investigated. The increasing amount of nano-Ca led to the improvement of the performance, however, the excessive amount of calcium has a detrimental effect on the biogas production, as when Ca concentration is 10 g/L, the result is even worse than in the case of the control experiment.

Mechanochemical processing of eggshell waste still finds new applications spheres and as both mechanochemistry and eggshell waste can be considered sustainable and applicable for the protection of the environment, it is highly probable that this area will further expand in the future.

### Limitations

Although the ball milling process is very beneficial in broadening the application scope of eggshell waste, it suffers from the traditional disadvantages of mechanochemistry, namely the contamination of the treated material with small fragments from milling balls and chambers and large temperature elevation at the contact points between the milled material and the milling balls. The latter phenomenon can lead to partial decarbonation of eggshell (it was already shown before that the decomposition temperature decreases with milling; Petkova et al., 2017), which might significantly alter the properties of the final product. However, in many cases, the production of CaO is necessary anyway, so it might also be beneficial. With regards to the eggshell membrane, the intensive milling degrades the soft fiber structure and can deteriorate adsorption properties (Baláž et al., 2016). However, upon proper tuning of the milling conditions (e.g., by using mild conditions when treating eggshell membrane), it is possible to avoid this undesirable phenomenon.

## Conclusion

Nature provides us with a lot of useful materials and despite the fact that some of them might seem to be without further use, their hidden application potential is always being discovered. This also accounts for the eggshell waste, one of the most common food wastes. Rather than being discarded on the landfills, it can be very useful in a rich plethora of applications. This review aimed to show its applications in catalysis, electrochemistry, therapeutics and after proper treatment by an environmentally harmless mechanochemistry, the scope of its applications can be further broadened. A large number of publications on this topic has been published in the last 2–3 years, which clearly shows that eggshell waste has recently become a very interesting material for various research groups around the world.

## Author Contributions

MB proposed the main idea and wrote the introductory part, a chapter devoted to the mechanochemical treatment of eggshell waste and conclusions. DR and EB wrote the part dealing with biomedical applications of the ES and ESM. DR-P and RL wrote the part devoted to the use of eggshell catalysis of bioactive compounds preparation, photocatalysis, and other environmentally beneficial applications. SP wrote the part devoted to the catalysis of biofuels using eggshell waste. TM wrote the part devoted to electrochemical applications of eggshell. All authors contributed to the article and approved the submitted version.

## Conflict of Interest

DR was employed by the company Mezomax Inc., San-Francisco, CA, USA. The remaining authors declare that the research was conducted in the absence of any commercial or financial relationships that could be construed as a potential conflict of interest.

## Footnotes

^1^PTH (parathyroid hormone). Its secretion is determined chiefly by serum ionized calcium concentration through negative feedback. PTH is secreted when Ca^2+^ concentration is decreased. RANK (Receptor activator of nuclear factor kappa-B); RANKL (Receptor activator of nuclear factor kappa-B ligand)—a membrane protein, a member of the tumor necrosis factor (TNF) cytokine family, it binds to RANK on cells of the myeloid lineage and functions as a key factor for osteoclast differentiation and activation; OPG (osteoprotegerin).

^2^Characters in the abbreviations encoding the amino acid residues: E – Glutamic acid, S – Serine, Y – Tyrosine, H – Histidine, L – Leucine, P – Proline, R – Arginine, M – Methionine, F - Phenylalanine, A – Alanine, W - Tryptophan, Q - Glutamine.

^3^www.mechsustind.eu CA18112: Mechanochemistry for Sustainable Industry. Available online at: www.mechsustind.eu (accessed September 30, 2020).

^4^www.Imamechanochemical.Com/About-Us/ International Mechanochemical Association. Available online at: www.imamechanochemical.com/about-us/ (accessed September 30, 2020).

## List of Abbreviations

APD: ammonium phosphate dibasic
API: active pharmaceutical ingredient
4-AP: 4-aminophenol
AV: Aloe vera
BG: bioactive glass
BMD: bone mineral density
CB: cuttlefish bone
CESM: carbonized eggshell membrane
CN: carbon nanostructure
CNC: cellulose nanocrystal
CREMP: cysteine-rich eggshell membrane protein
CTGF: connective tissue growth factor
2D: two-dimensional
3D: three-dimensional
ePTFE: expanded polytetrafluorethylene
ES: eggshell
ESM: eggshell membrane
ESMP: eggshell membrane powder
ESP: eggshell powder
FTIR: Fourier-transform infra-red
GEM: graphene-layered ESM
HA: hydroxyapatite
HUVEC: human umbilical vein endothelial cells
IL-1β: α-interleukin-1 beta
LFS: Lithium iron orthosilicate, Li_2_FeSiO_4_
MC: mineral composite
MS: mussel shell
NGC: nerve guidance channel
NIR: near infra-red
NP: nanoparticles
4-NP: 4-nitrophenol
OM: organic matrix
OPG: osteoprotegerin
ORR: oxygen reduction reaction
PCL: poly (ε-caprolactone)
PEM: particalized eggshell membrane
PEI: polyethyleneimine
PEP: processed eggshell membrane powder
PLA: poly(lactic) acid
PLD: pulsed laser deposition
PMN: polymorphonuclear
PTH: parathyroid hormone
PVA: polyvinyl alcohol
PVC: polyvinyl chloride
RANK: Receptor activator of nuclear factor kappa-β
RANKL: Receptor activator of nuclear factor kappa-β ligand
SC: supercapacitor
SESM: soluble eggshell membrane
SF: silk fibroin
TCNF: (2,2,6,6-tetramethylpiperidin-1-yl)oxyl (TEMPO)-oxidized cellulose nanofibrils
TEM: transition electron microscopy
TEMPO: (2,2,6,6-tetramethylpiperidin-1-yl)oxyl
TNF: tumor necrosis factor
VOC: volatile organic compound
XRD: X-ray diffraction.
